# Temperamental Sensitivities Differentially Linked With Interest, Strain, and Effort Appraisals

**DOI:** 10.3389/fpsyg.2020.551806

**Published:** 2021-01-08

**Authors:** Anna Maria Rawlings, Anna Tapola, Markku Niemivirta

**Affiliations:** ^1^Department of Education, University of Helsinki, Helsinki, Finland; ^2^School of Applied Educational Sciences and Teacher Education, Faculty of Philosophy, University of Eastern Finland, Joensuu, Finland

**Keywords:** temperament, reward and punishment sensitivity, motivation, interest, motivational appraisals, ESEM, PLS, longitudinal

## Abstract

The present research examined the connections between temperament (*punishment sensitivity; interindividual reward sensitivity; intraindividual reward sensitivity*), students’ domain- and course-specific motivational appraisals (interest, strain, effort), and performance, in two studies. Study 1 explored the relationships between temperamental sensitivities, motivational appraisals, and task achievement among secondary students (*N* = 268) in the domain of mathematics, using Exploratory Structural Equation Modeling (ESEM) for the analyses. Study 2 was conducted longitudinally among upper-secondary students (*N* = 155) during a course in four key school subjects. Subject interest was included alongside the temperamental sensitivities as a predictor of course-specific motivation and course grades, and the data were analysed with Partial Least Squares Structural Equation Modeling (PLS-SEM). Previous achievement was controlled in both studies. The findings showed temperamental sensitivities to be differentially linked with motivational appraisals. Punishment sensitivity in Study 1, and interindividual reward sensitivity (sensitivity to reward dependent on others’ approval or attention) in Study 2 were found to have an effect on psychological strain. In both studies, interest and effort were predicted by intraindividual reward sensitivity (positive responsiveness to novelty and own successes). In Study 2, subject interest was a consistent predictor of higher course interest and lower strain. In both studies, connections were found between strain and lower performance. The findings suggest individual characteristics may predispose students to certain motivational experiences, and contribute to educational outcomes, in both domain and course contexts and across subject content.

## Introduction

Individuals are known to differ in their temperamental predisposition to focus on, assess, and interpret environmental cues as signifying either something personally beneficial or potentially harmful. This differential propensity, known as reward and punishment sensitivity (e.g., Rothbart and Hwang, 2005), influences individuals’ appraisals of their environment (Derryberry et al., 2003) and shapes emotional and behavioural responses and patterns, to the extent that the sensitivities are seen as important motivators of approach and avoidance behaviour (Rothbart and Hwang, 2005; Corr, 2013; Corr et al., 2013; see also, Hidi, 2016). Further, reward and punishment sensitivities have also been found associated with factors related to individual well-being (e.g., job satisfaction and involvement; Van der Linden et al., 2007; stress perceptions; Williams et al., 2014). They may, hence, arouse motivationally adaptive appraisals or trigger stress responses also in a learning environment, potentially affecting academic and well-being-related outcomes. However, although the importance of temperamental approach and avoidance tendencies in guiding motivation has long been recognised (Elliot and Covington, 2001), studies on the relationship between reward and punishment sensitivity and motivation in a learning context remain sparse. The present research aims to shed light on these connections, by examining the influence of reward and punishment sensitivities on students’ motivational appraisals.

Students’ achievement-related behaviour and motivation in a certain context can be partly traced back to their appraisals of and responses to the given situation; in the appraisal process, students evaluate and balance academic demands with their perceived ability to cope and the impact these demands may have on their subjective well-being (Lazarus and Smith, 1988; Boekaerts and Niemivirta, 2000). These appraisals may be academically supportive (e.g., interest, effort; Hidi and Renninger, 2006) or emotionally costly (e.g., stress, exhaustion; Tuominen-Soini et al., 2012), and they may also influence each other in both positive (e.g., interest predicting effort; Trautwein et al., 2015) and negative ways (e.g., failure in spite of effort resulting in psychological strain; Boekaerts and Niemivirta, 2000). Although research shows students’ appraisals to depend in part on their motivational tendencies (Tapola and Niemivirta, 2008) and domain knowledge or skills (Boekaerts, 1993), the role of more stable individual characteristics is less known. Since temperamental sensitivities influence our interpretations of the environment and situations, and especially the affective valence and motivational relevance of those, looking into the connections between temperament and motivational appraisals, both facilitating and impeding, seems salient.

### Reward and Punishment Sensitivities

Two basic systems are considered as underlying approach and avoidance dimensions of behaviour, namely, reward and punishment sensitivity (Slobodskaya and Kuznetsova, 2013). These sensitivities illustrate a central distinction of temperament, recognised in a number of similar or overlapping conceptualisations (see, e.g., Rothbart and Hwang, 2005), describing relatively stable tendencies to perceive, focus on, and approach appetitive (*reward sensitivity*), or avoid or withdraw from perceived or actual aversive (*punishment sensitivity*) environmental cues.

In neurobiological terms, reward and punishment sensitivities are described by the behavioural inhibition and behavioural approach system conceptualisation (BIS/BAS; Gray and McNaughton, 2003). The BIS is thought to be activated by goal conflicts which spark anxiety, such as threatening situations that cannot be avoided or escaped and must therefore be approached one way or another (Corr and Cooper, 2016), or situations in which there is an inclination to approach a possible reward while also perceiving potential danger (DeYoung, 2010). The BAS, fairly sparsely theorised until quite recently (Corr and Cooper, 2016), is described as “*a global approach system*” (Corr and McNaughton, 2012, p. 2347) that has in empirical studies (e.g., Carver and White, 1994; Corr and Cooper, 2016) been shown to comprise a number of sub-dimensions – in fact, considering BAS as a unidimensional construct has even been actively discouraged (Corr, 2016; Corr and Cooper, 2016; Krupić et al., 2016b). In recent research, the BAS is seen as including anticipatory pleasure and openness to new experiences that are perceived as potentially rewarding, positive emotional responses to and high-intensity pleasure derived from attained rewards, as well as persistence and impulsivity (Corr and Cooper, 2016; see also, Gomez et al., 2020). Also relevant in the context of reward and punishment sensitivity is the conceptualisation of temperament of Cloninger et al. (1993), in which harm avoidance describes the tendency for anticipatory worry, passive avoidance, fear of uncertainty, and shyness of unknown people, and the reward-oriented dimensions include responding to novelty with exploration and strong approach reactions to cues of reward, social attachment and dependence on others’ approval, and perseverance in spite of frustration or fatigue.

Reward and punishment sensitivities are considered to be independent of each other, universal, but with fairly enduring individual differences in their relative emphasis (Corr, 2002; Corr et al., 2013). Moreover, as people differ also in what they perceive as rewarding or threatening (Corr, 2013), the same stimulus potentially inciting quite differing motivational responses in different individuals (see, Corr et al., 2016), current research sees reward sensitivity as separable into dimensions defined by the source of reward. For example, while novelty (e.g., novel situations and social encounters) is often considered an important reward dimension, as some individuals are seen as temperamentally disposed to find it appealing and hence worth approaching, others may, instead, perceive it as threatening and respond with withdrawal or avoidance (Rothbart and Hwang, 2005; Corr and Cooper, 2016). The company or attention of other people may likewise be experienced as rewarding or aversive (Cloninger et al., 1993; Torrubia et al., 2001; Corr, 2013), although sensitivity to reward related to social interaction has not often been considered as a separate dimension (for exceptions, see, Colder et al., 2011; Rawlings et al., 2017, 2020b). Notably, neuropsychological research is increasingly recognising sensitivity to social reward (conceptualised as rejection or acceptance feedback and social approval) alongside or instead of tangible incentives, such as monetary rewards (e.g., Kujawa et al., 2014; Flores et al., 2015; Oumeziane et al., 2017), highlighting the importance of individual differences in this sensitivity. Given the social nature of the school environment (Darnon et al., 2012), it appears fruitful to consider the influence of sensitivity to this kind of reward on students’ motivational experiences in a learning context.

The multidimensional structure of reward sensitivity is theorised as being due to the complex human environment, in which different goals are potentially adaptive, thus giving rise to a “demand” for different, reward-related approach strategies (see, Krupić et al., 2016a). Dimensions of reward sensitivity have fairly typically been operationalised to include general reward responsiveness; enjoyment and seeking of novelty, fun, and sensations; particular sensitivity for reward related to social acceptance or success, such as attention and praise; and persistence or a drive for the attainment of the desired reward (Cloninger et al., 1993; Carver and White, 1994; Colder and O’Connor, 2004; Rothbart, 2007; see also, Corr and Cooper, 2016). Grounding on these studies, and as in our previous research (Rawlings et al., 2017), we consider reward sensitivity as comprising two main dimensions. *Interindividual reward sensitivity* describes sensitivity to reward dependent on the actions and attitudes of other people, such as praise and attention, and *intraindividual reward sensitivity* to reward derived from one’s own actions and inner states. This dimension has occasionally been empirically observed to separate further into subdimensions, namely, enjoyment of novel situations and encounters, and enjoyment of and enthusiasm over personal successes (Rawlings et al., 2017; see also, Colder et al., 2011). These subdimensions bear some resemblance to the BAS conceptualisation dimensions described as anticipatory, future-oriented pleasure from novelty and potential rewards, and pleasure derived from attained or immediate rewards, respectively (see, Corr and Cooper, 2016; Satchell et al., 2017; Gomez et al., 2020), which have been found to correlate differentially with educationally interesting constructs, for example, conscientiousness and implicit theories of intelligence (Satchell et al., 2017).

### Motivational Appraisals: Interest, Strain, Effort

Interest, strain, and effort reflect students’ classroom experiences with motivational value and may be academically supportive or maladaptive in themselves, but they also appear interconnected and, hence, as interacting in a mutually supportive or hindering motivational cycle. Furthermore, connections between them and temperament are likely, as we will discuss in more detail in Section “Connections Between Temperament and Motivational Appraisals”.

Interest refers to a motivational state characterised by heightened attention, concentration, and affect that becomes manifested in the relation between a person and the object of interest (Hidi, 2006). It is often conceptualised, on the one hand, as *individual interest*, a relatively stable predisposition to re-engage with the object of interest that is developed over repeated exposure and engagement, or as *situational interest*, a momentary state of interest experienced in relation to a certain content or activity (Hidi and Renninger, 2006), on the other. In reference to the present context, students’ interest in a specific subject domain (e.g., mathematics) is seen to reflect one form of individual interest, whereas course interest, being more contextually bound, rather represents a variation of situational interest (see, Harackiewicz et al., 2008). Although interest may be evoked by different environmental triggers, especially appraisals of novelty and complexity have been suggested as playing a role (Silvia, 2005).

Interest activates or coincides with certain cognitive-affective responses that are beneficial for learning and performance. By drawing one’s attention and triggering positive energy (Hidi, 2006), interest in a content or domain has been found to support effort (i.e., diligent and persistent work exerted in academic tasks, and trying one’s best; Trautwein and Lüdtke, 2007; Trautwein et al., 2015). Studies also show interest to predict such effort independently and in interaction with trait conscientiousness (Trautwein et al., 2015), and support persistence in spite of difficulties encountered (Hidi and Renninger, 2006). Despite these connections with various learning-enhancing processes, the effect of interest on performance or academic achievement has sometimes been inconsistent (Nuutila et al., 2020), weak (Marsh et al., 2005), or even non-existent (Tanaka, 2007; Tapola et al., 2013). Yet, some evidence shows positive changes (instead of the level) in interest to facilitate task performance (Niemivirta and Tapola, 2007).

In turn, the key role of effort in successful academic outcomes has been shown in numerous studies (Trautwein et al., 2009; Skinner et al., 2016; Lei et al., 2018). As students persist in and engage with the content of a task or learning material, the likelihood of successful performance outcomes increases (Bandura, 1997). In addition, effort has also been linked with competence beliefs (Trautwein et al., 2009). Conversely, if one’s efforts do not bring hoped-for results, experiences of excessive difficulty might arise (Boekaerts and Niemivirta, 2000), which, in turn, may induce stress (e.g., Lazarus and Folkman, 1984; Strelau, 2001) and hinder interest arousal.

Strain describes the stress response resulting from an individual evaluating or perceiving their environment or situation as exceeding their abilities or resources and, hence, experiencing it as threatening or impairing their well-being (Lazarus and Folkman, 1984). If prolonged, such context- or situation-specific experiences may cumulate into burnout, characterised by chronic exhaustion, cynicism, and sense of inadequacy (Salmela-Aro et al., 2009). Following this, we conceptualise strain as stress and experienced difficulty.

While interest and effort support each other (Xu, 2018), they are likely negatively connected with strain. As mental energy is a limited psychological resource (Baumeister et al., 2007; Vohs et al., 2012), in situations where strain and negative affect are activated, less energy will be available for other cognitive processes (e.g., problem-solving and learning). Especially in complex and difficult tasks, negative emotions (e.g., anxiety) also impede thought-action patterns by narrowing down the perceived repertoire and use of flexible learning strategies, and reduce interest and motivation to explore and gain knowledge, as well as self-regulation of learning (Pekrun and Stephens, 2015). Consequently, feelings of exhaustion resulting from a prolonged depletion of mental and physical resources are predictive of inferior achievement (Madigan and Curran, 2020). Thus, psychological strain clearly is a factor exposing students to impaired academic performance.

### Connections Between Temperament and Motivational Appraisals

As a process of assessing aspects of the environment as personally beneficial or harmful (Derryberry et al., 2003), sensitivity to reward or punishment appears a potential dispositional factor influencing students’ motivational appraisals in a learning context. Connections have been observed between students’ temperamental tendencies and their goals in an achievement context (e.g., Elliot and Thrash, 2002, 2010; Bjørnebekk and Diseth, 2010; Rawlings et al., 2017, 2020b), and, further, other studies have examined how those goals, in turn, are linked with students’ experiences of interest (Tapola et al., 2013), strain (Tuominen-Soini et al., 2008), and effort expenditure (Hornstra et al., 2017). However, previous research on potential direct links between temperament and motivational appraisals in learning contexts remains, at best, sparse.

Interest functions as an appetitive approach mechanism that triggers attention, encourages exploration of novel situations, and counteracts anxiety and wariness of new things (Silvia, 2017). It and the related tendency to exert effort may hence be more easily sparked in individuals temperamentally inclined to approach rewards and experience positive affect and responsiveness to novelty (see, Hidi, 2016), whereas being temperamentally disposed to focus on and avoid potential threats might hinder interest arousal, as attention may be diverted from the content and perhaps learning itself to concerns over one’s capacity to cope successfully.

Further, temperament has been considered a potential antecedent to stress (Strelau, 2001). Connections have been found between punishment sensitivity and stress proneness (Heponiemi et al., 2003), negative affect in achievement situations (Bjørnebekk, 2007), and stress perceptions and avoidant coping (Williams et al., 2014). The harm-avoidance temperament dimension (Cloninger et al., 1993) that resembles punishment sensitivity has also been found associated with higher anticipated and experienced levels of stress (Ravaja et al., 2006). Conversely, responsiveness to reward has been linked with higher well-being (Taubitz et al., 2015) and tendency for pleasant affect (Heponiemi et al., 2003). However, it should be noted that this lesser stress proneness might depend on the kind of reward to which an individual is sensitive. In our previous research (Rawlings et al., 2017, 2020b), the effects sensitivity to praise and attention exert on student motivation were found to bear some resemblance to the effects of punishment sensitivity, in that both were connected with increased focus on and concern over one’s performance relative to others, which, in turn, has been linked with higher levels of stress (Tuominen-Soini et al., 2008).

Grounding on these previous findings, to increase understanding of how individual dispositions that contribute to our interpretations of achievement-related situations in a given domain (e.g., mathematics) or context (e.g., English course) influence motivational appraisals relevant in terms of both performance and well-being, we report two studies inspecting the predictions of temperamental sensitivities on students’ interest, strain, and effort. These connections have, to our knowledge, not been studied before, although they have the potential of informing researchers and educators of possible sources of educationally adaptive and maladaptive responses to the learning environment. In Study 1, the relationships were examined in the domain of mathematics within a cross-sectional design, and in Study 2, the framework was applied with repeated measures in the context of courses in several school subjects.

## Study 1

Reward and punishment sensitivities influence individuals’ perceptions and appraisals of the environment, motivate approach/avoidance behaviour, and are in themselves linked with individual well-being in both positive and negative ways (Derryberry et al., 2003; Rothbart and Hwang, 2005; Van der Linden et al., 2007; Corr, 2013; Corr et al., 2013; Williams et al., 2014). Further, the sensitivities have also been found connected with motivational constructs, for example, goals (Elliot and Thrash, 2002, 2010; Bjørnebekk and Diseth, 2010; Rawlings et al., 2017, 2020b), which are in turn linked both with appraisals that may support learning and well-being, such as interest (Tapola et al., 2013) and effort (Hornstra et al., 2017), as well as those that may be emotionally costly, like psychological strain (Tuominen-Soini et al., 2008). Furthermore, some previous findings suggest direct links between aspects of temperamental sensitivities and these motivational appraisals (e.g., responsiveness to novelty and interest; Hidi, 2016; Silvia, 2017; punishment sensitivity and stress proneness; Heponiemi et al., 2003; reward responsiveness and higher well-being; Taubitz et al., 2015). Grounding on this, the present research explores potential direct connections between temperament and motivational appraisals, as they may influence the way individuals respond to the learning environment, and, hence, affect educationally important outcomes.

Study 1 was conducted within the domain of mathematics, a subject known to predict many educational outcomes (e.g., Gottfried et al., 2013) and in which the relationships between motivation and skills or performance have been found to be clearer than in other subjects (e.g., language arts; Bong et al., 2012). We examined, firstly, the relationships between temperamental reward and punishment sensitivities and domain-specific motivational appraisals (i.e., mathematics interest, strain, and effort), and secondly, how these sensitivities and appraisals may be linked with performance in a mathematics task, while controlling for previous achievement in mathematics.

Based on the theorising and previous empirical findings outlined in the introduction, we expected punishment sensitivity to be predictive of lower levels of mathematics interest and higher levels of mathematics strain, due to it being associated with a tendency to perceive and focus on potential threats in the environment and an aversion to novelty (Derryberry et al., 2003; Rothbart and Hwang, 2005). However, while punishment sensitivity has been associated with performance-avoidance goals and orientations (Elliot and Thrash, 2002, 2010; Bjørnebekk and Diseth, 2010; Rawlings et al., 2017, 2020b), suggesting a connection with decreased effort, there is also some evidence of an “approach-to-avoid” strategy (Elliot and Thrash, 2001), in other words, that temperamental avoidance tendencies may be connected with performance-approach goals. In practice, this might mean that students prone to punishment sensitivity may exert extra effort on tasks or schoolwork, in order to avoid the threat of failure. Hence, as the findings so far seem somewhat mixed, we are not setting an explicit assumption on this relationship.

Further, we assumed interindividual reward sensitivity to be negatively predictive of mathematics interest, positively of mathematics strain, and negatively of mathematics effort. While necessarily tentative, due to this reward dimension being so far rather rarely studied, these assumptions are based on interindividual reward sensitivity predicting both performance-approach and performance-avoidance goal orientations (Rawlings et al., 2017, 2020b). The concerns over the adequacy of one’s performance relative to others depicted by these goal orientations might induce ego-protection, stress, and task avoidance (Boekaerts, 1993; Boekaerts and Niemivirta, 2000), and these, in turn, could be actualised as lower interest and effort, and higher psychological strain. Conversely, we expected the intraindividual reward sensitivity dimensions to be positively predictive of mathematics interest and effort, given that these types of dimensions are characterised by enjoyment of novelty, responsiveness to reward derived from one’s own actions and inner states, and approach behaviour (Cloninger et al., 1993; Carver and White, 1994; Colder and O’Connor, 2004; Rothbart, 2007; Rawlings et al., 2017; see also, Corr and Cooper, 2016; Gomez et al., 2020).

Regarding the predictions by motivational appraisals, based on previous theorising (e.g., Baumeister et al., 2007; Vohs et al., 2012; Pekrun and Stephens, 2015) and empirical findings (Madigan and Curran, 2020) showing a relationship between negative affect, exhaustion, and impaired academic performance, we assumed strain to predict task performance negatively. Likewise based on previous studies showing a positive relationship between academic achievement and persistence in and engagement with a task or learning content (Trautwein et al., 2009; Skinner et al., 2016; Lei et al., 2018) that characterise effort, we expected mathematics effort to be positively predictive of task performance. In contrast, as previous findings on the relationship between interest and achievement are mixed, with some studies showing achievement to predict interest but not vice versa (Garon-Carrier et al., 2016) and others showing no relationship either way (Nuutila et al., 2018; Xu, 2018), we refrained from making strict assumptions on the relationship between mathematics interest and task performance. It is important to note that studies looking at the direct effects of temperament on performance or achievement, following either the approach/avoidance tendencies or reward/punishment sensitivity conceptualisations, are virtually missing, and even the ones providing some indirect indications of such relations are few or rely on teacher- and parent-ratings rather than self-ratings of temperament (see, Al-Hendawi, 2013). We therefore examine the potential relationships between temperament and task performance without any specific assumptions.

Finally, we expected previous achievement in mathematics to be positively predictive of task performance.

### Method

#### Participants and Procedure

Participants were students (*N* = 268, *M*_*age*_ = 14.34; girls 49.6%) from twenty classes in seven comprehensive schools around Southern Finland. During the spring term of the eighth grade, the students responded to a questionnaire measuring their temperamental sensitivities and mathematics-related interest, strain, and effort. A few weeks later, as a measure of their mathematics task performance, the students took a low-stakes mathematics test during a mathematics class. The students’ most recent mathematics grade was used as an indicator of previous achievement. Participation was voluntary, and parental consent was obtained.

#### Measures

##### Temperamental sensitivities

Four dimensions of temperamental sensitivities were measured (Rawlings et al., 2017). Likert-type scales of 1 (“*Not at all true*”) to 7 (“*Completely true*”) were used to rate punishment sensitivity (SP; 5 items, e.g., “*I avoid talking or performing in public (e.g., at lectures)*”; α = 0.74); interindividual reward sensitivity (SRinter; four items, e.g., “*I often do things just to be praised (e.g., by the teacher)*”; α = 0.67) and intraindividual reward sensitivity on two sub-dimensions, namely, enjoying and seeking novelty (SRNS; e.g., “*I find new things exciting*”; α = 0.74) and positive expressiveness and enthusiasm over personal successes (SRPE; e.g., “*I express my excitement and enjoyment openly, when I succeed at something”;* α = 0.70).

##### Motivational appraisals

Students’ *interest* in mathematics (eight items, e.g., “*I am interested in maths*”; α = 0.92) was measured using a scale compiled from items used in previous studies (Frenzel et al., 2010, four items; Gottfried, 1985, two items; Marsh et al., 2005, two items) with the aim of capturing the core elements of individual interest (personal value and importance, emotional enjoyment, desire for gaining and deepening one’s knowledge, re-engagement and willingness to spend resources; e.g., Hidi and Renninger, 2006; Renninger and Hidi, 2011; Hidi, 2016; see also Knogler, 2017). *Strain* (3 items, e.g., “*Studying maths really stresses me*”; α = 0.73) was operationalised in terms of indicators that reflect a challenge to students’ coping or well-being, such as difficulty, exhaustion, and stress, following the Lazarus and Folkman (1984, p. 19) definition of psychological stress, namely, “*a particular relationship between the person and the environment that is appraised by the person as taxing or exceeding his or her resources and endangering his or her well-being*”. Finally, three items measuring *effort* e.g., “*I always try to solve all problems we have for homework in math*”; α = 0.83) were adapted from Trautwein et al. (2009, one item; 2015, two items).

##### Performance measures

The students’ task performance was measured using a validated low-stakes, age-relevant, paper-and-pen mathematics test.

The students’ task performance was measured using a validated low-stakes, age-relevant, paper-and-pen mathematics test (Räsänen and Leino, 2005) consisting of arithmetical calculations and verbal and applied problems, with a maximum of 40 points (1 point for correct, 0 for incorrect or omitted answers; *M* = 21.02, *SD* = 6.51). A regular mathematics class of 45 min duration was allocated for the test, and while students could skip or leave individual problems unanswered, they were instructed to attempt to solve them all. The students were also told their task scores would not be given to their teachers and would not affect their grade.

Finally, the students’ most recent mathematics grades (in Finland, given on a scale of 4 “*Fail*” to 10 “*Distinction*”; *M* = 7.63, *SD* = 1.42), used as an indicator of previous achievement, were obtained from their teachers.

#### Analyses

The data were analysed using Mplus Statistical Software version 7.1 (Muthén and Muthén, 1998-2015). A model was specified in the Exploratory Structural Equation Modeling (ESEM; e.g., Marsh et al., 2014) framework, with Geomin rotation and Robust Maximum Likelihood (MLR) estimator. Temperamental sensitivities are considered to elicit behavioural effects in interaction with each other, and variables used for assessing them are therefore unlikely to be factorially pure (Corr and McNaughton, 2008). Due to this, constricting cross-loadings to zero as in confirmatory structural equation modeling (CFA-SEM) may be problematic, also as artificially suppressing cross-loadings not very close to zero may inflate latent correlations of factors (Morin et al., 2013; Marsh et al., 2014) and even result in misinterpretation of the relationships between phenomena. As ESEM includes many of the benefits of CFA-SEM, such as providing statistical criteria for evaluating different factor structures (e.g., significance tests and fit indices) whilst allowing for cross-loadings that may reflect the very nature of the phenomena under study, it was considered a suitable method of analysis for the present research (Mai et al., 2018)^1^. Temperament factors were specified as exploratory, and mathematics interest, strain, and effort, due to their conceptual separateness from each other, as confirmatory factors. Potential clustering across classes was taken into account by using the TYPE = COMPLEX specification, as implemented in Mplus software.

A model was specified and tested (see, Supplementary Figure 1), in which task performance was regressed on the motivational appraisals and temperamental sensitivities, and the motivational appraisals, in turn, on temperamental sensitivities, whilst controlling for the effects of previous achievement. Indirect effects on task performance from previous achievement (via temperamental sensitivities and motivational appraisals) and from temperamental sensitivities (via motivational appraisals) were also estimated. Regarding evaluating the goodness-of-fit of a model, the χ^2^ statistic is widely used (e.g., Hu and Bentler, 1999), but it is also known to be sensitive to, for instance, sample size or minor deviations from normality (e.g., Morin et al., 2013). Therefore, the χ^2^ statistic was complemented with the root mean square of error approximation (RMSEA; values < 0.08 seen as indicating acceptable, and < 0.06 as good fit to the data), comparative fit index (CFI; values > 0.90 considered as acceptable, and > 0.95 as excellent fit to the data), and standardised root mean squared residual (SRMR; recommended value < 0.08) (see, Hu and Bentler, 1999; Morin et al., 2013).

### Results

The initial model fit was acceptable [χ^2^(341, *N* = 268) = 628.667, *p* < 0.001; RMSEA = 0.056 (90% CI 0.049, 0.063); CFI = 0.903; SRMR = 0.055]. However, an inspection of the modification indices showed two suggested, substantively meaningful modifications, which were made iteratively. The residual correlation of two items (“*I like doing math things also in my spare time*”; “*I sometimes look at math-related web sites in my spare time*”) measuring interest in mathematics was freed, and one mathematics interest item (“*I enjoy challenging math tasks*”) was allowed to crossload negatively on the mathematics strain factor. After these modifications, the model fit the data well, χ^2^(339, *N* = 268) = 502.840, *p* < 0.001; RMSEA = 0.042 (90% CI 0.034, 0.050); CFI = 0.945; SRMR = 0.050. Latent correlations, as well as descriptive statistics and Cronbach’s α values (calculated from averaged sum scores for illustrative purposes using SPSS 25) are given in Table 1. Factor loadings of the temperamental sensitivity items and the motivational appraisal items are given in Supplementary Appendix A.

**TABLE 1:** Descriptive statistics, Cronbach’s alpha values, and latent correlations (Study 1, *N* = 268).

Variable (scale)	*M (SD)*	α	*1*	*2*	*3*	*4*	*5*	*6*	*7*	*8*
1 Punishment sensitivity (1–7)	3.89 (1.29)	0.74	–							
2 Interindividual reward sensitivity (1–7)	3.06 (1.08)	0.67	−0.02	–						
3 Intraindividual reward sensitivity (NS) (1–7)	4.25 (1.19)	0.74	−0.38	0.21	–					
4 Intraindividual reward sensitivity (PE) (1–7)	4.62 (1.40)	0.70	−0.21	0.31	0.31	–				
5 Mathematics interest (1–7)	3.22 (1.41)	0.92	−0.05	0.13	0.32	0.03	–			
6 Mathematics strain (1–7)	3.65 (1.45)	0.73	0.16	0.06	−0.10	0.02	−0.51	–		
7 Mathematics effort (1–7)	4.59 (1.56)	0.83	−0.10	0.04	0.29	0.02	0.76	−0.40	–	
8 Task performance (0–40)	21.02 (6.51)	–	0.02	0.04	0.12	−0.05	0.40	−0.52	0.33	
9 Previous achievement (4–10)	7.63 (1.42)	–	0.13	0.05	0.11	0.01	0.55	−0.59	0.47	0.65

*NS, novelty-seeking; PE, positive expressiveness. Descriptive statistics and Cronbach’s alpha reliabilities of temperamental sensitivities and motivational appraisals calculated from averaged sum scores for illustrative purposes. Correlations ≥ |0.12| significant at *p* < 0.05.*

**FIGURE 1:**
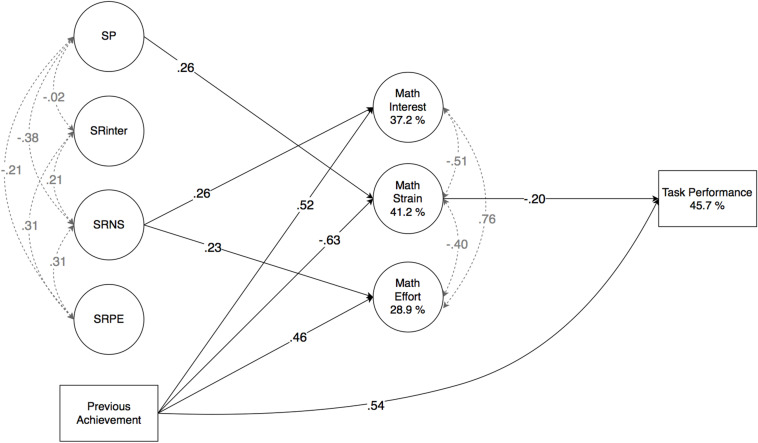
Results of ESEM analysis (*N* = 268; Study 1) illustrated. For clarity, factor loadings of observed variables are omitted, and only significant (*p* < 0.05) effects (β) are reported. SP, punishment sensitivity; SRinter, interindividual reward sensitivity; SRNS, intraindividual reward sensitivity (novelty-seeking); SRPE, intraindividual reward sensitivity (positive expressiveness).

Significant direct effects are illustrated in Figure 1. As expected, SP predicted mathematics strain positively (β = 0.26, *p* = 0.015), but remained unrelated to interest. Against expectations, SRinter did not predict any variable significantly. SRNS predicted mathematics interest and effort (β = 0.26, *p* = 0.001, and β = 0.23, *p* = 0.032, respectively), as expected, but SRPE did not. Previous achievement predicted task performance (β = 0.54, *p* < 0.001), as assumed, as well as mathematics interest (β = 0.52, *p* < 0.001) and effort (β = 0.46, *p* < 0.001) positively, and strain negatively (β = −0.63, *p* < 0.001). Of the motivational appraisals, mathematics strain predicted task performance (β = −0.20, *p* = 0.008), as assumed, but mathematics effort did not. In addition, indirect predictions were also found. Previous achievement predicted task performance indirectly via mathematics strain (β = 0.13, *p* = 0.015), and a small indirect effect from SP on task performance via strain (β = −0.05, *p* = 0.056) was also observed.

Accounting for clustering across classes did not change the estimates, as all effects remained identical. The model explained 37.2% (*p* < 0.001) of the variance in mathematics interest, 41.2% (*p* < 0.001) of the variance in mathematics strain, 28.9% (*p* < 0.001) of the variance of mathematics effort, and 45.7% (*p* < 0.001) of the variance in task performance. All direct effects are given in Table 2.

**TABLE 2:** Direct effects and explained variance of dependent variables (Study 1, *N* = 268).

	Mathematics interest		Mathematics strain		Mathematics effort		Task performance	
				
Predictor	β	*p*	β	*p*	β	*p*	β	*p*
Previous achievement	0.52	0.00	−0.63	0.00	0.46	0.00	0.54	0.00
Punishment sensitivity	−0.03	0.58	0.26	0.02	−0.09	0.34	−0.02	0.76
Interindividual reward sensitivity	0.07	0.34	0.08	0.38	−0.01	0.93	0.04	0.58
Intraindividual reward sensitivity (NS)	0.26	0.00	0.04	0.73	0.23	0.03	0.05	0.55
Intraindividual reward sensitivity (PE)	−0.09	0.31	0.05	0.66	−0.08	0.41	−0.08	0.23
Mathematics interest							0.00	0.99
Mathematics strain							−0.20	0.01
Mathematics effort							−0.03	0.83
*R*^2^	0.37		0.41		0.29		0.46	

*NS, novelty-seeking; PE, positive expressiveness. All *R*^2^*p* < 0.001.*

### Discussion

Study 1 examined the connections between eighth-graders’ temperamental reward and punishment sensitivities and their motivational appraisals (interest, strain, effort) in the domain of mathematics, and the links of temperament and motivational appraisals with the students’ performance in a mathematics task, while controlling for their previous achievement. We expected punishment sensitivity to be predictive of lower levels of mathematics interest and higher levels of mathematics strain; interindividual reward sensitivity to be negatively predictive of mathematics interest, positively of mathematics strain, and negatively of mathematics effort; the intraindividual reward sensitivity dimensions to be positively predictive of mathematics interest and effort; previous achievement in mathematics to be positively predictive of task performance; and mathematics strain to be negatively, and effort to be positively predictive of task performance. The results were partly in line with our assumptions.

As expected, punishment sensitivity was related to higher strain, in line with previous findings linking it with stress (Williams et al., 2014; see also, Ravaja et al., 2006) and negative affect (Bjørnebekk, 2007), as well as more broadly with research suggesting temperament may be an antecedent to stress (Strelau, 2001). However, contrary to our expectations, it remained unrelated to interest. To some extent, this could be seen as echoing previous results, in which punishment sensitivity has been associated with concerns over one’s performance relative to others as depicted by the performance-avoidance goal orientation, but not with the academic disinterest inherent in the work-avoidance goal orientation (Rawlings et al., 2017, 2020b). Further, punishment sensitivity has often been found unrelated to mastery strivings (Rawlings et al., 2017, 2020b; Farrell and Walker, 2019), which, in turn, are linked with interest (Tapola et al., 2013). In other words, proneness to punishment sensitivity does not, in and of itself, necessarily preclude interest, although the tendency to want to avoid negative outcomes associated with punishment sensitivity (Farrell and Walker, 2019) might hamper it in the long run, particularly if anxiety and stress levels grow high.

Contrary to our expectations, interindividual reward sensitivity remained almost entirely unrelated to the motivational appraisals, apart from a small positive correlation with interest. It may be that students at this educational stage do not perceive academic engagement as something that impacts their social relations and standing either positively or negatively, and hence interindividual reward sensitivity would not be an important contributing factor to either the positive (interest, effort) or negative (strain) motivational responses examined here. In future research, it might be useful to explore the connections between interindividual reward sensitivity and other constructs emphasising the instrumental value of studying and performing well (e.g., utility value, Eccles, 2009).

Intraindividual reward sensitivity with the tendency for novelty-seeking was positively connected with both interest and effort, as assumed and in keeping both with previous findings linking interest and responding to novelty with exploration (Silvia, 2017), and the suggestion that interest might be more easily sparked in individuals given to approach tendencies and responsiveness to novelty (Hidi, 2016). However, the positive-expressive dimension of intraindividual reward sensitivity was unconnected with the motivational appraisals. Interestingly, in previous research (Rawlings et al., 2017), this dimension has also had fewer connections with students’ goal orientations compared with the other temperament dimensions, only being negatively related to the goal orientations associated with performance concerns. While the novelty-seeking dimension could be seen as describing positive emotional responses and a “drive” to approaching a (potential) reward, the positive-expressive dimension is more related to the pleasure experienced when reward has been attained (see, Gomez et al., 2020). By the present results, this tendency to delight in one’s achievements appears less predictive of students’ motivation.

Unsurprisingly, previous achievement in mathematics was a strong predictor of task performance, but also of mathematics interest and effort, whereas contrary to our expectations, effort remained unrelated to task achievement. Significant bivariate correlations were observed between mathematics interest and effort and task achievement, and there is a possibility that some kind of suppression effect may be the reason they did not manifest as significant predictions^2^. However, the observed results do confirm some previous findings of achievement predicting interest (Garon-Carrier et al., 2016) and effort (Marsh et al., 2016), rather than vice versa. Furthermore, previous achievement was also negatively predictive of strain, and strain, as expected, predicted task performance negatively. This result parallels previous studies, for example, on anxiety (e.g., Pekrun et al., 2017) and school burnout (Paloş et al., 2019; Madigan and Curran, 2020), and calls also for acknowledging the possibility of reciprocal relations between students’ strain-related responses and academic achievement. In other words, weakness in academic skills may induce strain experiences, which, in turn, interfere with students’ cognitive and motivational resources during demanding performance situations. Accordingly, it is worth noting that besides the detrimental effect of strain on students’ engagement during performance, low performance of students high in strain may also reflect deficits in their basic academic skills (skills deficit model) or study strategies (Pekrun and Stephens, 2015; Pekrun et al., 2017).

The findings add to the understanding of how temperament may be connected with motivation in a learning context. The importance of considering sensitivity to qualitatively different rewards separately is underlined by the differences in their respective linkages (or lack thereof) with motivational appraisals. However, the role of punishment sensitivity appears particularly worthy of note, given its connection with mathematics strain and the negative link between strain and task performance observed here, as well as the detrimental effects of strain on well-being more generally. As punishment sensitivity was not, against our expectations, related to decreased interest, and as strain and interest were negatively linked, enabling and supporting interest experiences and development might be a means of buffering students prone to punishment sensitivity against strain. Providing an emotionally safe classroom climate for exploring novel learning situations and content, in which aspects likely to arouse anxiety in students prone to punishment sensitivity are controlled or minimised (e.g., lack of pressure to “perform” in front of others; ensuring a suitable, not excessive level of difficulty; avoiding direct, public comparison of students’ skills), may further assist in decreasing strain, and support positive learning experiences for all students, regardless of their temperamental sensitivities. An increased understanding of temperamental tendencies may enhance educators’ pedagogical sensitivity and ability to respond to their students’ individual characteristics and needs.

## Study 2

In Study 2, a similar framework of temperamental sensitivities predicting motivational appraisals was applied to a course-specific context with repeated measures in four subjects (Finnish, Swedish, English, advanced syllabus level mathematics). This replication of the design enabled us to inspect the extent to which the relationships might be more general, or specific to a course in a given subject. Further, examining the relationships over the duration of four different courses allowed us to investigate also the stability of and dynamics between the motivational appraisals over time.

The study was conducted directly after the transition from comprehensive to upper-secondary education.^3^ In upper-secondary schools, the school year is organised modularly into five to six periods, with students choosing five to six courses per period. The duration of each period and, hence, course is about 1.5 months (Finnish National Agency for Education, 2018). After completing the general upper-secondary school (usually, in 3–4 years), the students sit a national, standardised matriculation examination; some subjects in both the exam and the general upper-secondary curriculum are compulsory, others the students may choose. Finnish, Swedish, and English are all compulsory subjects, but while studying mathematics is also mandatory, students choose either basic or advanced syllabus level, and in this, mathematics differs from the other subjects included in this study. The courses examined in the present study can, hence, be said to represent key subjects in the curriculum.

In Study 2, subject interest was included as a predictor of course-specific appraisals in each course, as it is often considered as facilitating motivation in a context (e.g., arousing positive affect, supporting situational interest being triggered; Hidi and Renninger, 2006; promoting effort exertion; Trautwein et al., 2015; Xu, 2018). Furthermore, in order to capture students’ subjective success experiences, their satisfaction in their course grade was also included as a dependent variable. The assumptions regarding the relationships between the variables were made based on previous theorising and empirical research, as outlined in the “Introduction” Section and in the assumptions of Study 1. We note that while differences in the settings of the studies reported here may contribute to some differences in predictions, we do not assume certain connections to be tied to a given context, either generally in relation to a subject domain (i.e., mathematics) or course-specifically in various subject domains (i.e., Finnish, Swedish, English, mathematics). Hence, our assumptions are, to the extent they are supported by previous research, the same as in Study 1.

Punishment sensitivity was expected to be predictive of lower levels of course interest and higher levels of course strain. Interindividual reward sensitivity was assumed to be predictive of lower course interest, higher course strain, and lower course effort, whereas intraindividual reward sensitivity was expected to be predictive of higher course interest and course effort. Further, we expected subject interest to be positively predictive of course interest, and both subject and course interest to be positively predictive of course effort and negatively of course strain. Finally, we expected course strain to be negatively, and previous achievement and course effort positively predictive of course grade, and course grade, in turn, to predict students’ satisfaction in their course performance. We did not expect these effects to differ substantively between the different subjects.

### Method

#### Participants and Procedure

The participants were the whole age-cohort of first-year students (*N* = 172; due to absence, effective *N* = 155; age 16–17 years; girls 56.1%) attending the only general upper-secondary school of a medium-sized, middle-class, mainly industrial town in Central Finland, comprising a fairly typical sample of youths from similar, non-metropolitan Finnish towns with a socio-economically relatively homogenous, almost entirely native Finnish population. The study utilised the data collected from the students attending the first course in four key subjects: Finnish (*N* = 140), Swedish (*N* = 124), English (*N* = 141), and advanced-level mathematics (*N* = 81).

Data collection for Study 2 was intense, with a maximum of 13 data points per participating student (note that not every participant took part in every course). The study being conducted among the whole age-cohort of first-year students attending the only general upper-secondary school in town enabled reducing contextual variation and the impact of external factors, as school practices and culture, and, in many cases, teachers in a given subject remained the same for all participants.

Participation was voluntary, consent was obtained, and confidentiality was assured.

#### Measures

In the beginning of the school year, participants rated their punishment sensitivity (five items), interindividual reward sensitivity (four items), and two dimensions of intraindividual reward sensitivity (novelty seeking, three items; possessive expressiveness, two items) on the scale (Rawlings et al., 2017) described in more detail in Study 1. At this point, participants also rated their interest in each examined subject on a scale of 1 (“*Not at all interesting*”) to 7 (“*Extremely interesting*”). Final comprehensive school grades (scale 4 “*Fail*” – 10 “*Distinction*”) in the subjects, retrieved from school records, were used as an indicator of previous achievement (Finnish *M* = 8.33, *SD* = 0.86; Swedish *M* = 8.05, *SD* = 1.13; English *M* = 8.30, *SD* = 1.05; mathematics *M* = 8.77, *SD* = 0.76). A visualisation of the data collection process is included in supplemental materials (Supplementary Figure 2).

To enable examining the stability, interrelationships, and outcomes of students’ motivational appraisals, two measurement points were established during the first course in each subject under study (t1 early part of the course; t2 immediately following the course exam). On Likert-type scales from 1–7, participants rated their course-specific interest (three items, e.g., *“The content of this course is/has been interesting”*), strain (stress and experienced difficulty of the course; four items, e.g., *“This course is/has been stressful for me”*), and effort (three items, e.g., *“I am putting/have put a lot of effort into this course”*). Note that while the motivational appraisal items resemble those used in Study 1, they were worded so as to suit a course-specific context. Further, their overall number was kept small, in order to keep the questionnaire compact and to interfere with studying as little as possible. Finally, after the course and having received the course grade, participants rated their satisfaction in their performance (“*Do you feel you reached the goals you had set yourself? Did you do as well as you expected to?”*; single dichotomous item with *No* = 1, *Yes* = 2). Course grades, also measured on a scale of 4 “*Fail*” – 10 “*Distinction*”, were retrieved from school records and used as an indicator of course achievement.

#### Analyses

Analyses were run separately for each subject examined. The data were analysed using Partial Least Squares structural equation modeling (PLS-SEM). PLS-SEM is a viable alternative to covariance-based SEM in the case of more exploratory studies (Hair et al., 2014), and when the model is complex and the sample relatively small (Sarstedt et al., 2017), due to it imposing less strict distributional assumptions on data (Sanchez, 2013). Given the complexity of our model and the data size, PLS-SEM was seen as the methodological option most suited to the study at hand. We used the “plspm” package (Sanchez et al., 2015) with R software version 3.2.3, with a centroid weighting scheme for estimating inner weights, and a bootstrapping procedure with 500 bootstrap samples for estimating parameter significance. The “missForest” package (Stekhoven, 2013) was used to impute missing values. Overall, the percentage of these was low, with a range from 0.07% (English) to 0.18% (Finnish) in temperamental sensitivities and motivational appraisals, but in the course satisfaction variable, attrition was higher, ranging from 5.7% (Finnish) to 29.0% (Swedish). Data for the course satisfaction variable were collected at the beginning of the next course in each subject, and missing value analysis showed the attrition observed in this variable to be due to non-participation in this second course during the year of data collection. Further auxiliary analyses testing the selectiveness of this attrition showed no significant group differences in course achievement between the students from whom course satisfaction data was available, and those from whom it was not.

PLS-SEM includes a measurement and a structural model, which we evaluated following Hair et al. (2014). As PLS-SEM prioritises the indicators according to their individual reliability, composite reliability is recommended for examining the internal consistency of the measurement model, with values 0.60–0.70 seen as acceptable in exploratory stages of research. Convergent validity is established using indicator loadings, which should be significant and greater than 0.7 on the intended latent factors, and the average variance extracted (AVE), which should be above 0.5. Discriminant validity is assessed by examining indicator cross-loadings, which should not be greater than loadings on the intended factors, and using the so-called Fornell–Larcker criterion, whereby the square root of the AVE of each construct should be greater than its correlation with any other construct. It should here be noted that as indicator loadings < 0.7 are often observed in social sciences, recommended practice is to remove items with loadings between 0.40–0.70 only if this would increase the construct’s composite reliability or AVE above the threshold values. Finally, significance of path coefficients and explained variance (*R*^2^) are the most important criteria for examining the validity of the structural model.

#### Specifying and Testing the Model

A measurement model of the temperamental sensitivity and motivational appraisal items loading on their hypothesised latent variables was specified for all subjects. Initially, a temperament model comprising SP and the three reward sensitivity dimensions (SRinter, SRNS, SRPE), as in Study 1, was tested. Three indicators (one SP “*I prefer to withdraw in situations that feel unpleasant or difficult*”; one SRinter “*I often aim to impress other people*”; one SRNS “*I think it is exciting to get into new and surprising situations*”) were removed iteratively, due to insignificant loading, low AVE, and/or poor communality. Strong cross-loadings were then found between indicators intended to measure the two expected intraindividual reward sensitivity dimensions (i.e., novelty-seeking; positive expressiveness). Therefore, a parsimonious model with SP (four items) and the two main reward sensitivity dimensions of the original conceptualisation (see, Rawlings et al., 2017), namely, interindividual (SRinter; three items) and intraindividual (SRintra; four items, two each representing the NS and PE dimensions), was tested. (For items retained in the measurement model and their outer loadings on latent variables, see, Supplementary Appendix B).

The internal consistency of this measurement model was evaluated using the Dillon–Goldstein (DG) ρ measure of composite reliability, with values ranging between 0.76 and 0.86 for the temperament variables, and between 0.83 and 0.92 for the motivational appraisals. Convergent validity was established using outer loadings and AVE. Outer loadings ranged between 0.54 and 0.85 for temperamental sensitivity items, and between 0.55 and 0.93 for motivational appraisal items. Three items had some individual non-significant loadings in some subjects (see, Supplementary Appendix B, but as these were considerably higher (≥ 0.55) than the suggested cut-off point of 0.40 and all AVE were > 0.50, the items were retained within the latent variables, as suggested by Hair et al. (2014). Finally, cross-loadings and the square root of AVE were examined, to establish discriminant validity. All cross-loadings were smaller than loadings on intended factors. The square root of AVE was greater than correlations with other constructs, with the exception of the two measurement points for strain (in Swedish and English) and effort (in English). This was considered as unproblematic with regard to discriminant validity, given these represented measurements of the same construct at two different points in time. As the two-dimensional reward sensitivity was both theoretically and substantively meaningful and in line with the original conceptualisation, and as internal consistency and convergent and discriminant validity could be established, this measurement model was seen as describing the data adequately. Note that as we examined the factor structure of temperament variables as well as predictive relationships separately for each subject, and as the students participating in a particular course differ slightly from one subject to another, there is slight variation in the item loadings on their respective temperament factors between the four subjects examined. Descriptive statistics, DGρ, and AVE of variables are given in Table 3.

**TABLE 3:** Descriptive statistics, composite reliabilities, and average variance extracted of latent factors (Study 2).

Variable (scale)	Finnish (*N* = 140)			Swedish (*N* = 124)			English (*N* = 141)			Mathematics (*N* = 81)		
				
	*M (SD)*	*DG*ρ	*AVE*	*M (SD)*	*DG*ρ	*AVE*	*M (SD)*	*DG*ρ	*AVE*	*M (SD)*	*DG*ρ	*AVE*
SP (1–7)	3.60 (1.30)	0.84	0.539	3.45 (1.27)	0.83	0.506	3.50 (1.29)	0.84	0.536	3.64 (1.35)	0.84	0.611
SRinter (1–7)	2.74 (0.98)	0.76	0.516	2.76 (1.05)	0.80	0.575	2.77 (1.04)	0.79	0.549	2.58 (1.01)	0.79	0.582
SRintra (1–7)	4.45 (1.11)	0.85	0.571	4.52 (1.13)	0.86	0.603	4.47 (1.10)	0.85	0.581	4.34 (1.02)	0.85	0.537
Subject interest (1–7)	4.30 (1.53)			4.10 (1.82)			5.31 (1.41)			5.68 (1.18)		
Previous achievement (4–10)	8.33 (0.86)			8.05 (1.13)			8.30 (1.05)			8.77 (0.76)		
Course interest t1 (1–7)	4.58 (1.08)	0.91	0.761	3.97 (1.22)	0.89	0.856	4.53 (1.13)	0.87	0.690	4.95 (1.12)	0.87	0.763
Course strain t1 (1–7)	3.11 (0.86)	0.81	0.523	3.52 (1.11)	0.87	0.785	3.02 (1.21)	0.89	0.665	3.25 (1.35)	0.89	0.709
Course effort t1 (1–7)	4.99 (1.06)	0.86	0.664	4.86 (1.11)	0.85	0.803	4.89 (1.13)	0.83	0.580	5.29 (1.04)	0.83	0.556
Course interest t2 (1–7)	4.85 (0.99)	0.89	0.731	4.49 (1.09)	0.91	0.873	4.80 (1.09)	0.92	0.788	4.91 (1.15)	0.92	0.775
Course strain t2 (1–7)	3.06 (0.98)	0.86	0.596	3.37 (1.14)	0.90	0.829	3.11 (1.32)	0.92	0.752	3.51 (1.34)	0.92	0.728
Course effort t2 (1–7)	4.94 (1.12)	0.86	0.676	4.75 (1.20)	0.87	0.826	4.79 (1.29)	0.87	0.680	4.97 (1.20)	0.87	0.588
Course grade (4–10)	7.70 (1.04)			7.20 (1.57)			7.55 (1.47)			7.78 (1.47)		

*Means and standard deviations calculated from averaged sum scores for illustrative purposes. SP, punishment sensitivity; SRinter, interindividual reward sensitivity; SRintra, intraindividual reward sensitivity.*

Regarding the structural model, course satisfaction was regressed on course grade, and both on all preceding variables; t2 motivational appraisals (interest, strain, effort) were regressed on t1 appraisals and the antecedents (temperament, subject-specific interest, previous achievement); and t1 appraisals were regressed on the antecedents.

### Results

**FIGURE 2:**
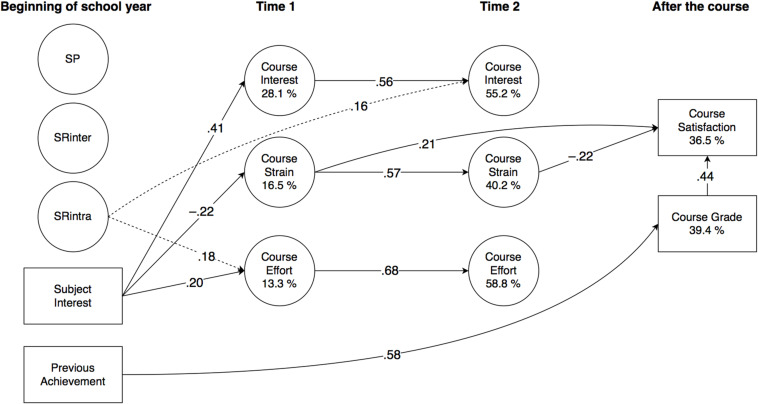
Results of PLS-SEM analysis (Finnish, *N* = 140; Study 2) illustrated. For clarity, factor loadings of observed variables and correlations of latent variables are omitted, and only significant (*p* < 0.05) effects (β) are reported. (Dashed lines represent standardised effects at *p* < 0.10.) SP, punishment sensitivity; SRinter, interindividual reward sensitivity; SRintra, intraindividual reward sensitivity.

**FIGURE 3:**
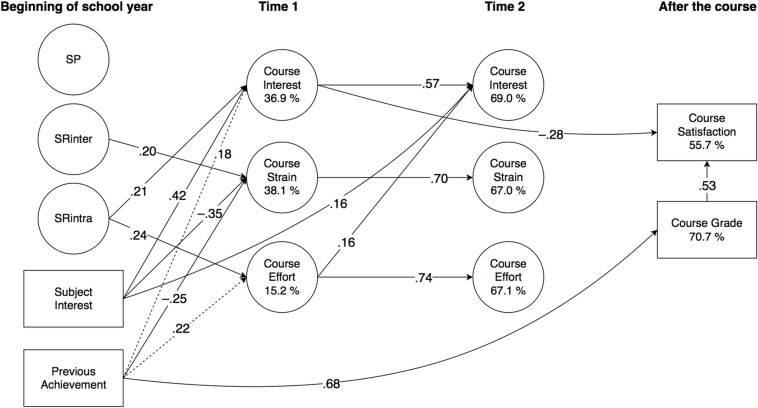
Results of PLS-SEM analysis (Swedish, *N* = 124; Study 2) illustrated. For clarity, factor loadings of observed variables and correlations of latent variables are omitted, and only significant (*p* < 0.05) effects (β) are reported. (Dashed lines represent standardised effects at *p* < 0.10.) SP, punishment sensitivity; SRinter, interindividual reward sensitivity; SRintra, intraindividual reward sensitivity.

Significant effects are detailed in the subchapters below, and illustrated in Figures 2 (Finnish), Figures 3 (Swedish), 4 (English), and 5 (mathematics). All direct effects are given in Tables 4 (Finnish), 5 (Swedish), 6 (English), and 7 (mathematics), with significance levels indicated with 95% confidence intervals (CI). Correlations are given in Supplementary Appendix B, with the square root of AVE in the diagonal.

**FIGURE 4:**
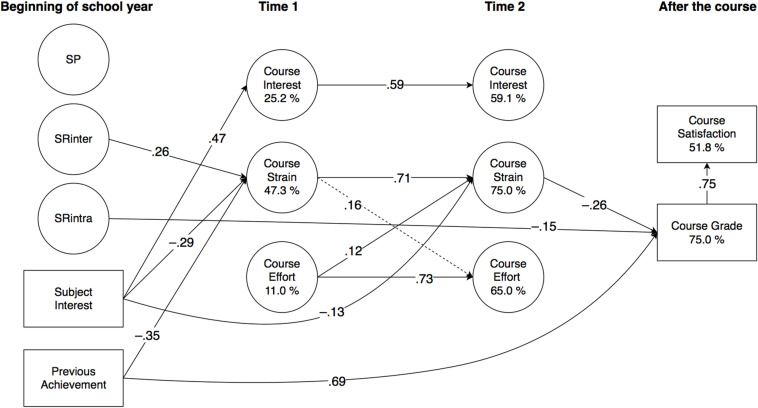
Results of PLS-SEM analysis (English, *N* = 141; Study 2) illustrated. For clarity, factor loadings of observed variables and correlations of latent variables are omitted, and only significant (*p* < 0.05) effects (β) are reported. (Dashed lines represent standardised effects at *p* < 0.10.) SP, punishment sensitivity; SRinter, interindividual reward sensitivity; SRintra, intraindividual reward sensitivity.

**FIGURE 5:**
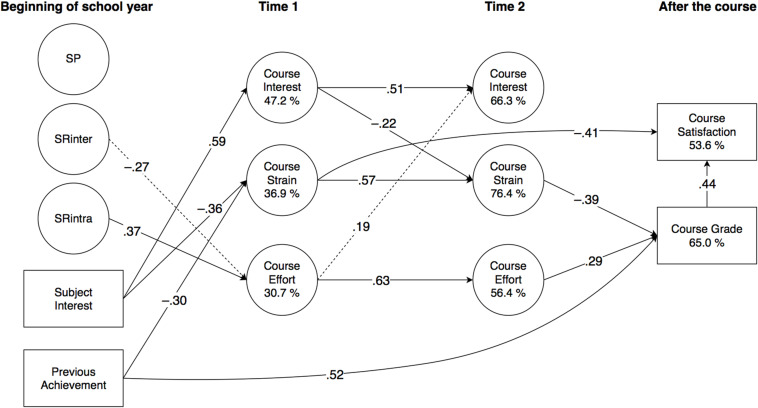
Results of PLS-SEM analysis (Mathematics, *N* = 81; Study 2) illustrated. For clarity, factor loadings of observed variables and correlations of latent variables are omitted, and only significant (*p* < 0.05) effects (β) are reported. (Dashed lines represent standardised effects at *p* < 0.10.) SP, punishment sensitivity; SRinter, interindividual reward sensitivity; SRintra, intraindividual reward sensitivity.

**TABLE 4:** Direct effects and explained variance of dependent variables (Finnish, *N* = 140; Study 2).

Predictor	Course interest t1		Course strain t1		Course effort t1		Course interest t2		Course strain t2		Course effort t2		Course grade		Course satisfaction	
								
	β	95% CI	β	95% CI	β	95% CI	β	95% CI	β	95% CI	β	95% CI	β	95% CI	β	95% CI
SP	−0.09	(−0.27, 0.10)	0.10	(−0.24, 0.37)	−0.02	(−0.23, 0.20)	0.04	(−0.13, 0.21)	0.08	(−0.19, 0.29)	0.01	(−0.17, 0.19)	0.05	(−0.17, 0.25)	−0.09	(−0.28, 0.10)
SRinter	−0.03	(−0.22, 0.17)	0.17	(−0.04, 0.36)	−0.06	(−0.27, 0.17)	−0.04	(−0.19, 0.13)	0.08	(−0.10, 0.25)	0.10	(−0.11, 0.27)	−0.06	(−0.23, 0.12)	0.01	(−0.20, 0.21)
SRintra	0.13	(−0.06, 0.32)	0.02	(−0.22, 0.29)	0.18	(−0.01, 0.38)	0.16	(−0.00, 0.32)	0.03	(−0.16, 0.22)	0.10	(−0.08, 0.28)	−0.01	(−0.17, 0.16)	−0.11	(−0.30, 0.08)
Subject interest	0.41	(0.27, 0.55)	−0.22	(−0.39, −0.06)	0.20	(0.00, 0.37)	0.12	(−0.04, 0.25)	−0.14	(−0.31, 0.07)	0.00	(−0.13, 0.13)	−0.04	(−0.18, 0.11)	0.03	(−0.13, 0.22)
Previous achievement	0.07	(−0.09, 0.23)	−0.07	(−0.22, 0.11)	0.03	(−0.15, 0.20)	−0.02	(−0.13, 0.10)	0.03	(−0.14, 0.20)	0.07	(−0.07, 0.19)	0.58	(0.46, 0.72)	0.05	(−0.13, 0.25)
Course interest t1							0.56	(0.39, 0.72)	0.19	(−0.02, 0.40)	−0.00	(−0.19, 0.18)	−0.17	(−0.40, 0.09)	−0.06	(−0.29, 0.17)
Course strain t1							−0.10	(−0.26, 0.09)	0.57	(0.45, 0.70)	−0.07	(−0.23, 0.09)	−0.02	(−0.20, 0.19)	0.21	(0.02, 0.40)
Course effort t1							0.03	(−0.11, 0.16)	−0.01	(−0.16, 0.16)	0.68	(0.53, 0.81)	0.11	(−0.14, 0.33)	0.13	(−0.10, 0.36)
Course interest t2													0.08	(−0.26, 0.14)	−0.07	(−0.25, 0.11)
Course strain t2													−0.06	(−0.24, 0.11)	−0.22	(−0.41, −0.03)
Course effort t2													−0.03	(−0.30, 0.23)	−0.06	(−0.25, 0.14)
Course grade															0.44	(0.29, 0.57)
*R*^2^	0.28		0.17		0.13		0.55		0.40		0.59		0.39		0.37	

*SP, punishment sensitivity; SRinter, interindividual reward sensitivity; SRintra, intraindividual reward sensitivity; CI, confidence interval. All *R*^2^ significant at 95% CI.*

**TABLE 5:** Direct effects and explained variance of dependent variables (Swedish, *N* = 124; Study 2).

Predictor	Course interest t1		Course strain t1		Course effort t1		Course interest t2		Course strain t2		Course effort t2		Course grade		Course satisfaction	
								
	β	95% CI	β	95% CI	β	95% CI	β	95% CI	β	95% CI	β	95% CI	β	95% CI	β	95% CI
SP	0.06	(−0.12, 0.25)	0.03	(−0.21, 0.26)	0.04	(−0.19, 0.25)	−0.08	(−0.19, 0.04)	−0.01	(−0.12, 0.11)	−0.07	(−0.22, 0.08)	0.10	(−0.05, 0.23)	−0.10	(−0.27, 0.07)
SRinter	0.05	(−0.13, 0.22)	0.20	(0.03, 0.38)	−0.15	(−0.36, 0.07)	0.05	(−0.11, 0.19)	0.06	(−0.07, 0.19)	−0.07	(−0.22, 0.10)	0.06	(−0.09, 0.21)	−0.02	(−0.19, 0.15)
SRintra	0.21	(0.04, 0.36)	−0.09	(−0.27, 0.07)	0.24	(0.03, 0.43)	0.05	(−0.11, 0.20)	0.06	(−0.08, 0.20)	0.09	(−0.04, 0.23)	−0.09	(−0.24, 0.06)	−0.07	(−0.25, 0.11)
Subject interest	0.42	(0.21, 0.60)	−0.35	(−0.52, −0.15)	0.04	(−0.22, 0.27)	0.16	(0.02, 0.28)	0.03	(−0.13, 0.18)	0.01	(−0.13, 0.15)	0.09	(−0.07, 0.23)	0.00	(−0.17, 0.16)
Previous achievement	0.18	(−0.01, 0.38)	−0.25	(−0.42, −0.11)	0.22	(−0.01, 0.43)	−0.09	(−0.22, 0.05)	−0.12	(−0.28, 0.02)	0.04	(−0.09, 0.19)	0.68	(0.55, 0.82)	0.08	(−0.16, 0.32)
Course interest t1							0.57	(0.40, 0.73)	−0.09	(−0.25, 0.08)	0.05	(−0.16, 0.24)	−0.03	(−0.24, 0.17)	−0.28	(−0.55, −0.05)
Course strain t1							−0.13	(−0.27, 0.02)	0.70	(0.58, 0.81)	0.04	(−0.11, 0.21)	−0.00	(−0.20, 0.19)	0.02	(−0.20, 0.23)
Course effort t1							0.16	(0.01, 0.31)	0.08	(−0.06, 0.21)	0.74	(0.60, 0.87)	0.01	(−0.21, 0.24)	0.09	(−0.19, 0.35)
Course interest t2													0.03	(−0.17, 0.19)	0.14	(−0.13, 0.42)
Course strain t2													−0.11	(−0.31, 0.07)	−0.21	(−0.45, 0.02)
Course effort t2													0.01	(−0.18, 0.20)	0.06	(−0.17, 0.33)
Course grade															0.53	(0.30, 0.75)
*R*^2^	0.37		0.38		0.15		0.69		0.67		0.67		0.71		0.56	

*SP, punishment sensitivity; SRinter, interindividual reward sensitivity; SRintra, intraindividual reward sensitivity; CI, confidence interval. All *R*^2^ significant at 95% CI.*

**TABLE 6:** Direct effects and explained variance of dependent variables (English, *N* = 141; Study 2).

Predictor	Course interest t1		Course strain t1		Course effort t1		Course interest t2		Course strain t2		Course effort t2		Course grade		Course satisfaction	
								
	β	95% CI	β	95% CI	β	95% CI	β	95% CI	β	95% CI	β	95% CI	β	95% CI	β	95% CI
SP	−0.07	(−0.28, 0.16)	0.07	(−0.11, 0.27)	−0.05	(−0.25, 0.22)	0.06	(−0.10, 0.21)	−0.04	(−0.15, 0.08)	−0.10	(−0.23, 0.05)	−0.02	(−0.17, 0.11)	−0.05	(−0.21, 0.10)
SRinter	0.02	(−0.17, 0.19)	0.26	(0.09, 0.43)	0.02	(−0.26, 0.28)	0.04	(−0.11, 0.18)	0.02	(−0.09, 0.13)	−0.07	(−0.22, 0.09)	0.02	(−0.09, 0.13)	0.03	(−0.13, 0.18)
SRintra	0.02	(−0.16, 0.21)	0.02	(−0.13, 0.16)	0.10	(−0.14, 0.30)	0.13	(−0.04, 0.29)	0.03	(−0.06, 0.14)	0.04	(−0.11, 0.19)	−0.15	(−0.27, −0.05)	−0.07	(−0.24, 0.10)
Subject interest	0.47	(0.28, 0.65)	−0.29	(−0.47, −0.10)	0.10	(−0.16, 0.36)	0.11	(−0.06, 0.30)	−0.13	(−0.26, −0.01)	0.01	(−0.14, 0.16)	0.09	(−0.07, 0.23)	0.00	(−0.17, 0.16)
Previous achievement	−0.03	(−0.22, 0.17)	−0.35	(−0.52, −0.18)	−0.06	(−0.30, 0.22)	0.07	(−0.08, 0.23)	−0.05	(−0.18, 0.10)	0.00	(−0.17, 0.15)	0.69	(0.59, 0.78)	−0.15	(−0.44, 0.11)
Course interest t1							0.59	(0.39, 0.78)	−0.03	(−0.12, 0.07)	0.05	(−0.12, 0.22)	0.09	(−0.06, 0.23)	0.02	(−0.16, 0.20)
Course strain t1							−0.10	(−0.25, 0.05)	0.71	(0.58, 0.82)	*0.16*	(−*0.01, 0.32)*	0.09	(−0.08, 0.26)	0.05	(−0.19, 0.32)
Course effort t1							0.06	(−0.10, 0.21)	0.12	(0.00, 0.22)	0.73	(0.57, 0.85)	−0.07	(−0.22, 0.08)	0.06	(−0.17, 0.28)
Course interest t2													−0.00	(−0.16, 0.14)	0.01	(−0.14, 0.18)
Course strain t2													−0.26	(−0.43, −0.07)	−0.08	(−0.34, 0.18)
Course effort t2													0.07	(−0.07, 0.20)	−0.12	(−0.34, 0.09)
Course grade															0.75	(0.49, 0.99)
*R*^2^	0.25		0.47		0.11		0.59		0.75		0.65		0.75		0.52	

*SP, punishment sensitivity; SRinter, interindividual reward sensitivity; SRintra, intraindividual reward sensitivity; CI, confidence interval. All *R*^2^ significant at 95% CI.*

**TABLE 7:** Direct effects and explained variance of dependent variables (Mathematics, *N* = 81; Study 2).

Predictor	Course interest t1		Course strain t1		Course effort t1		Course interest t2		Course strain t2		Course effort t2		Course grade		Course satisfaction	
								
	β	95% CI	β	95% CI	β	95% CI	β	95% CI	β	95% CI	β	95% CI	β	95% CI	β	95% CI
SP	−0.15	(−0.36, 0.11)	0.08	(−0.20, 0.33)	0.05	(−0.22, 0.37)	0.05	(−0.12, 0.20)	−0.08	(−0.22, 0.12)	−0.18	(−0.38, 0.03)	0.09	(−0.07, 0.27)	0.04	(−0.22, 0.31)
SRinter	−0.06	(−0.25, 0.19)	0.15	(−0.11, 0.39)	−0.27	(−0.50, 0.01)	−0.08	(−0.29, 0.13)	0.04	(−0.10, 0.20)	0.07	(−0.17, 0.31)	0.02	(−0.09, 0.13)	0.03	(−0.13, 0.18)
SRintra	0.07	(−0.16, 0.30)	0.03	(−0.24, 0.31)	0.37	(0.05, 0.68)	0.12	(−0.14, 0.39)	0.10	(−0.10, 0.30)	−0.02	(−0.28, 0.31)	0.04	(−0.17, 0.26)	−0.14	(−0.40, 0.15)
Subject interest	0.59	(0.38, 0.76)	−0.36	(−0.55, −0.15)	0.08	(−0.20, 0.40)	0.17	(−0.02, 0.35)	−0.02	(−0.17, −0.14)	0.12	(−0.19, 0.39)	−0.02	(−0.23, 0.21)	0.03	(−0.20, 0.27)
Previous achievement	−0.03	(−0.22, 0.16)	−0.30	(−0.48, −0.09)	−0.11	(−0.48, 0.32)	−0.11	(−0.29, 0.03)	−0.25	(−0.37, 0.14)	0.04	(−0.20, 0.30)	0.52	(0.33, 0.69)	0.03	(−0.28, 0.30)
Course interest t1							0.51	(0.30, 0.69)	−0.22	(−0.40, −0.05)	0.00	(−0.28, 0.29)	−0.01	(−0.27, 0.24)	−0.20	(−0.53, 0.11)
Course strain t1							−0.03	(−0.24, 0.17)	0.57	(0.43, 0.71)	0.00	(−0.33, 0.32)	0.15	(−0.15, 0.43)	−0.41	(−0.75, −0.08)
Course effort t1							0.19	(−0.01, 0.41)	0.02	(−0.19, 0.25)	0.63	(0.26, 0.89)	−0.05	(−0.42, 0.27)	0.16	(−0.14, 0.51)
Course interest t2													−0.06	(−0.30, 0.18)	0.08	(−0.20, 0.35)
Course strain t2													−0.39	(−0.69, −0.05)	0.05	(−0.36, 0.47)
Course effort t2													0.29	(0.04, 0.59)	−0.08	(−0.37, 0.23)
Course grade															0.44	(0.16, 0.73)
*R*^2^	0.47		0.37		0.31		0.66		0.76		0.56		0.65		0.54	

*SP, punishment sensitivity; SRinter, interindividual reward sensitivity; SRintra, intraindividual reward sensitivity; CI, confidence interval. All *R*^2^ significant at 95% CI.*

As a general outline, there were both similarities and differences between the domains. Briefly, regarding the predictions of the temperament factors, SP remained separate from all other variables in all subjects, against expectations. SRinter was, as expected, positively related to course strain in Swedish and English, and negatively to course effort in mathematics. Likewise as assumed, SRintra was positively related to course interest and effort in Finnish and Swedish, and to course effort in mathematics, but unexpectedly, it also negatively predicted course grade in English. Further, in all subjects, subject interest predicted course interest positively and course strain negatively, and previous achievement positively predicted course grade, which, in turn, positively predicted course satisfaction. In all subjects apart from Finnish, previous achievement was also a negative predictor of course strain.

The model significantly explained the variance of all dependent variables. At t1, explained variance in interest ranged from 25.2% (English) to 47.2% (mathematics); in strain from 16.5% (Finnish) to 47.3% (English); and in effort from 11.0% (English) to 30.7% (mathematics). At t2, explained variance in interest ranged from 55.2% (Finnish) to 69.0% (Swedish); in strain from 40.2% (Finnish) to 76.4% (mathematics); and in effort from 56.4% (mathematics) to 67.1% (Swedish). For course grade, explained variance ranged from 39.4% (Finnish) to 75.0% (English), and for course satisfaction, from 36.5% (Finnish) to 55.7% (Swedish).

#### Predictive Effects on Motivational Appraisals

Against expectations, SP remained unconnected with all other variables. As expected, SRinter predicted strain at t1 in Swedish (β = 0.20) and English (β = 0.26), and had a small negative effect on effort at t1 in mathematics (β = −0.27; 95% CI −0.50, 0.01). Also in line with our expectations, SRintra predicted interest at t1 in Swedish (β = 0.21), and effort at t1 in Swedish and in mathematics (β = 0.24; β = 0.37, respectively). A small effect on effort at t1 and an increase in interest at t2 in Finnish was also observed (β = 0.18; 95% CI −0.01, 0.38; β = 0.16, 95% CI −0.00, 0.32, respectively).

As expected, in all subjects, subject interest predicted interest at t1 positively and strain at t1 negatively, with the effects on interest ranging from β = 0.41 in Finnish to β = 0.59 in mathematics, and on strain from β = −0.22 in Finnish to β = −0.36 in mathematics. Subject interest also predicted effort at t1 in Finnish (β = 0.20), an increase in interest at t2 in Swedish (β = 0.16), and a decrease in strain at t2 in English (β = −0.13). Previous achievement was a negative predictor of strain at t1 in all subjects apart from Finnish, with the effects ranging from β = −0.25 in Swedish to β = −0.35 in English.

#### Stability and Interrelationships of Motivational Appraisals

All motivational appraisals showed significant stability in all subjects. In interest, the autoregressive effect ranged from β = 0.51 in mathematics to β = 0.59 in English; in strain from β = 0.57 in Finnish and mathematics to β = 0.71 in English; and in effort from β = 0.63 in mathematics to β = 0.74 in Swedish.

Although the motivational appraisals remained fairly independent of each other, some significant predictions were observed. As expected, interest at t1 predicted a decrease in strain at t2 in mathematics (β = −0.22). Effort at t1 predicted an increase in interest at t2 in Swedish (β = 0.16). A small effect from effort at t1 on interest at t2 was also observed in mathematics (β = 0.19; 95% CI −0.01, 0.41). In English, effort at t1 predicted an increase in strain at t2 (β = 0.12), and there was also a small reciprocal effect from strain at t1 to an increase in effort at t2 (β = 0.16; 95% CI −0.01, 0.32).

#### Predictive Effects on Course Outcomes

In all subjects, previous achievement predicted the course grade, ranging from β = 0.52 in mathematics to β = 0.69 in English, and course grade predicted course satisfaction, ranging from β = 0.44 in Finnish and mathematics to β = 0.75 in English. Unexpectedly, the course grade was also negatively predicted by SRintra in English (β = −0.15), and also, as assumed, by strain at t2 in English and in mathematics (β = −0.26 and β = −0.39, respectively), as well as positively by effort at t2 in mathematics (β = 0.29). In addition, there were also predictions on course satisfaction by the motivational appraisals that had not been specified in our assumptions, so that interest at t1 predicted course satisfaction negatively in Swedish (β = −0.28), whereas in Finnish, it was predicted positively by strain at t1 (β = 0.21), but negatively by strain at t2 (β = −0.22). Finally, in mathematics, strain at t1 predicted course satisfaction negatively (β = −0.41).

#### Indirect Effects

SRinter had an indirect effect on strain at t2 in Swedish (β = 0.18) and English (β = 0.21). SRintra had a positive, indirect effect on interest at t2 and effort at t2 in Finnish (β = 0.24 and β = 0.22, respectively) and Swedish (β = 0.21 and β = 0.27, respectively), but negative on course grade and course satisfaction in English (β = −0.16 and β = −0.20, respectively). In all subjects, subject interest had an indirect effect on interest at t2 (β_Finnish_ = 0.37; β_Swedish_ = 0.45; β_English_ = 0.42; β_mathematics_ = 0.49) and strain at t2 (β_Finnish_ = −0.19; β_Swedish_ = −0.25; β_English_ = −0.34; β_mathematics_ = −0.36). Likewise in all subjects, previous achievement had an indirect effect on course grade (β_Finnish_ = 0.57; β_Swedish_ = 0.72; β_English_ = 0.73; β_mathematics_ = 0.68) and course satisfaction (β_Finnish_ = 0.28; β_Swedish_ = 0.50; β_English_ = 0.40; β_mathematics_ = 0.42), and on strain at t2 in all subjects apart from Finnish (β_Swedish_ = −0.30; β_English_ = −0.31; β_mathematics_ = −0.44). Finally, strain at t1 had an indirect effect on course satisfaction in mathematics (β = −0.42). All indirect effects, with significance levels indicated with 95% CI, are given in Supplementary Appendix B.

### Discussion

Study 2 examined the influence of temperamental reward and punishment sensitivities and subject interest on students’ course-specific interest, strain, and effort (i.e., motivational appraisals), and how these factors relate to course achievement and students’ satisfaction in it, during the first course in four subjects in general upper-secondary school, while controlling for previous achievement. We expected punishment sensitivity to be predictive of lower course interest and higher course strain; interindividual reward sensitivity to be predictive of lower course interest and effort and higher course strain; intraindividual reward sensitivity to be predictive of higher course interest and effort; subject interest to predict course interest, and both subject and course interest to predict course strain negatively and effort positively; and course strain to be negatively, and previous achievement and course effort positively predictive of course grade, and course grade, in turn, to predict students’ satisfaction in their performance.

While overall, there were fewer effects than anticipated, and although different courses displayed some differences in the effects, there were also similarities across the courses, and the observed connections were generally in line with our expectations and reflected previous work. Further, in spite of the differences in contexts, there were also some similarities between the relationships observed here, and those found in Study 1. However, punishment sensitivity not predicting any motivational appraisals was against expectations and somewhat surprising, although the non-significant relation with interest is in line with the findings of Study 1. As to the lack of connection with strain, it should be remembered that novelty is considered to be an important cue activating the anxiety associated with punishment sensitivity. It may be that at this upper-secondary-school stage of students’ educational paths, situations within the learning context are no longer new to them, and punishment sensitivity might hence be less likely to become activated. Furthermore, some students may have learned productive coping strategies (Evans et al., 2018) or developed effective self-regulation (Scrimin et al., 2018), which have been found to compensate for punishment sensitivity. We note also that punishment sensitivity correlated positively with course grade in Swedish, and negatively with later course effort in English and mathematics. While interpreting the former of these is difficult without further data, a relationship with withdrawal of effort is suggestive of the avoidance behaviour associated with this sensitivity, and one might speculate the non-significance of these effects to be due to sample size.

Interindividual reward sensitivity was consistently positively correlated with course strain, and intraindividual reward sensitivity, in turn, with course interest in three out of four subjects and with course effort in all subjects, reflecting our expectations of these relationships. However, not all of these correlations manifested as significant effects. In two of the four subjects examined, interindividual reward sensitivity was, as expected, directly and indirectly related to strain, although not to lower interest or effort. While this aspect of reward sensitivity has been little studied in the field of motivation, the connections with strain reinforce existing findings of its associations with motivationally less supportive outcomes (higher work avoidance; concerns over one’s performance relative to others; lower mastery strivings; Rawlings et al., 2017, 2020b). Proneness to this sensitivity may guide students to emphasise gaining and maintaining social approval instead of, or through, schoolwork and learning, and this kind of attentional bias (see, Derryberry et al., 2003) may render them more vulnerable to increased stress and experienced difficulty. This might especially be the case, if academic ability and achievement are valued within one’s social environment – as they are likely to be in the upper-secondary context – and therefore, also perceived as important ways of gaining others’ praise and attention. It might also be noted that the relationship with strain was significant in Swedish and English, in other words, the two non-native languages. As speaking the language is generally required in class, these are the subjects in which students are most likely to have to “expose” publicly their abilities and, moreover, their inabilities, and consequently, in a sense, themselves. However, it appears that in the upper-secondary-school context, students’ desire for gaining public praise and recognition does not necessarily undermine their interest and effort, as there were no negative predictions from interindividual reward sensitivity on these motivational appraisals.

In line with our assumptions and the results of Study 1, intraindividual reward sensitivity had direct and/or indirect links with higher interest and/or effort in three of the four subjects examined, hence appearing motivationally more beneficial. This is in keeping with the understanding that interest is related to a positive responsiveness to novelty (Hidi and Renninger, 2006; Silvia, 2017), as well as previous research linking this sensitivity with mastery-oriented goal strivings (Rawlings et al., 2017), which are, in turn, associated with interest (Tapola et al., 2013) and effort (Hornstra et al., 2017).

The expected positive predictions from subject interest to course interest, and negative to course strain, were observed in all subjects. Furthermore, course interest and course strain were negatively correlated in all subjects, and in mathematics, earlier interest predicted lower later strain. The results are in line with numerous previous studies showing how students’ pre-existing interest in a domain facilitates the triggering of more context- or situation-specific feelings of interest (Tapola et al., 2013; Fryer et al., 2016). At the same time, interest seems to diminish the probability of high strain, which is also in line with both theoretical notions (e.g., Hidi and Renninger, 2006) and previous studies (e.g., Song et al., 2019) suggesting interest-driven activities to be experienced as less strenuous and costly than when interest is lacking. The expected prediction from subject interest on course effort was only observed in Finnish, and against expectations, course interest did not predict course effort in any subject. However, while we did not observe the reciprocal predictions found by Xu (2018) between interest and effort, our correlational connections are similar in that the correlations between course effort and both subject and course interest were quite consistently positive, supporting the notion that interest and effort are likely to co-occur.

The course grade was, as expected, strongly predicted by previous achievement in the subject. However, against our expectations and previous empirical findings (Trautwein et al., 2009; Skinner et al., 2016; Lei et al., 2018), effort was only connected with the course grade in one of the four courses examined, namely, mathematics. It may be that the impact of effort on achievement can be more readily detected in a subject in which there is relatively little variance in students’ initial skill levels, as is the case here with the students who opted for advanced syllabus level mathematics. Then again, it may also be that students’ appraisals of their effort reflect experiences and activities that do not directly translate into course performance. Behavioural measures of effort might provide some insights into the matter. Strain predicted the course grade negatively in two subjects, in line with our expectations and reinforcing the suggestion that negative emotions and feelings of difficulty may impede processes and strategies important for learning (Ho and Guthrie, 2013; Pekrun and Stephens, 2015). We also note that while previous achievement only predicted course interest in Finnish, their correlation was positive in Swedish and English, and in these latter two subjects, course interest, in turn, correlated positively with course grade.

Finally, students’ satisfaction in their performance was found to have a mostly straightforward and expected relationship with course achievement – the better a student achieved, the more satisfied they were with their performance, whereas experiencing the course as stressful and difficult appeared mainly to reduce satisfaction.

## General Discussion

The present research reported two studies examining the connections between students’ temperamental sensitivities, motivational appraisals of interest, strain, and effort, and performance. Study 1 was conducted within the domain of mathematics among eighth-graders, and Study 2 over the duration of a course in four key subjects in the first year of general upper-secondary school. The findings of both studies were partially in line with our expectations.

In Study 1, punishment sensitivity was, as expected, connected with higher psychological strain, reflecting previous findings linking it with higher stress perceptions and stress levels (Ravaja et al., 2006; Williams et al., 2014), although against expectations not with lower interest or effort. Further against our expectations, interindividual reward sensitivity did not predict any of the motivational appraisals. The effects of intraindividual reward sensitivity, however, were partly as assumed, as the tendency for novelty-seeking was positively linked with domain interest and effort, while positive expressiveness remained unconnected with the motivational appraisals. The effects of novelty-seeking are in keeping with previous theorising about the links between responsiveness to novelty and interest (Hidi and Renninger, 2006; Silvia, 2017) as well as findings linking intraindividual reward sensitivity with academically more adaptive phenomena (e.g., mastery-oriented goal strivings; Rawlings et al., 2017) that, in turn, have been found associated with interest (Tapola et al., 2013) and effort (Hornstra et al., 2017).

In Study 2, punishment sensitivity was not related to strain, and indeed remained quite separate from all other variables. Instead, interindividual reward sensitivity was more in line with our assumptions, as it predicted higher strain in Swedish and English, had a small negative effect on effort in mathematics, and correlated negatively with interest and effort in all subjects. This reflects previous findings, in which interindividual reward sensitivity has appeared academically more maladaptive (e.g., lower mastery strivings, higher concerns over one’s relative performance, higher work avoidance; Rawlings et al., 2017, 2020b). Intraindividual reward sensitivity, which in Study 2 was represented as a single factor, had direct and/or indirect effects on interest, effort, or both in three out of four subjects examined. Intraindividual reward sensitivity, thus, appears to be motivationally mostly adaptive,

Further regarding Study 2, in line with our assumptions, subject interest facilitated course interest and buffered students against strain in all school subjects, and in mathematics, also interest earlier in the course predicted decreased strain at the end of the course, reinforcing the view of interest as motivationally adaptive (Hidi and Renninger, 2006). However, contrary to our expectations as well as previous findings (Hidi and Renninger, 2006; Trautwein et al., 2015), the role of subject as well as course interest in predicting course effort remained quite minor, with a prediction by subject interest significant only in Finnish, in spite of positive bivariate correlations observed in all subjects except English. This sparseness of predictions from interest to effort may to some extent reflect the nature of upper-secondary studies: apart from advanced-level mathematics, the subjects examined here are compulsory, and students must exert some effort in order to pass, whether interested or not. Instead, effort early on in the course supported later interest in Swedish, in line with Xu (2018).

In considering the results, it is of importance to note the difference in the respective contexts of the two studies. Study 1 was conducted in reference to one domain with a time lag between self-evaluations and task performance only, and Study 2 in a course context on several school subjects with a more intensive repeated measures design. The role of situational factors, known to affect students’ motivational appraisals (see, e.g., Rauthmann et al., 2016), is quite likely to be more pronounced in the course context of Study 2 than in Study 1, where predictions were examined between relatively stable ‘individual difference factors’ (i.e., temperament and domain-specific motivational appraisals). The differential context may partly explain what we consider the most important difference between the predictions of the two studies, namely, that domain-level (i.e., more stable) strain was related to punishment sensitivity in Study 1, whereas in Study 2, course-level (i.e., more contextual) strain was predicted by interindividual reward sensitivity, itself strongly related to (perceptions of) the social context. Furthermore, the differences may in some part be traced back to the age and stage of the participating students, in that academic achievement may be socially more highly valued and hence more important among upper-secondary students than in comprehensive school. It may also be that older, on the whole perhaps more academically oriented students have developed effective coping or self-regulation methods that compensate for punishment sensitivity (see, Evans et al., 2018; Scrimin et al., 2018). Examining these possibilities requires both longitudinal research and designs in which situational influences, and their interplay with temperamental sensitivities, are taken into account.

However, also similarities were observed between the two studies. As regards temperament, intraindividual reward sensitivity was found to relate positively with interest and effort in both domain and course contexts, thus appearing less influenced by environmental or situational factors. This seems fairly straightforward and meaningful, as the dimension describes sensitivity to reward derived from one’s own actions and inner states (Cloninger et al., 1993; Carver and White, 1994; Colder and O’Connor, 2004; Rothbart, 2007; Rawlings et al., 2017), and would hence by definition be less likely to be affected by contextual factors. Moreover, in spite of some significant, positive correlations, no effects from (subject or course) interest to (task or course) achievement were found in either study, reflecting similar findings in previous research (Garon-Carrier et al., 2016; Nuutila et al., 2018; Xu, 2018). While it is possible that the strong prediction by previous achievement left relatively little variance to be explained, strain did predict lower task performance in Study 1, and lower course grades in two out of four subjects in Study 2. Finally, it should be noted that the role of previous achievement was overall somewhat more important than expected, as it predicted not only task performance in Study 1 and respective course grades in all subjects in Study 2, but also mathematics interest and effort in Study 1, and effort in Swedish in Study 2. Achievement predicting interest and effort, rather than vice versa, is also in line with the findings of Garon-Carrier et al. (2016) and Marsh et al. (2016), respectively. However, we note that in the advanced syllabus level course of mathematics in Study 2, effort did predict course grade. It may be that the impact of effort on achievement is more detectable in situations in which there is relatively little variation in students’ initial skill levels. Further, both in Study 1 and in three out of four subjects in Study 2, previous achievement was negatively related to strain, which, in a sense, echoes the consistent findings of negative connections between achievement and burnout (Paloş et al., 2019; see also, Madigan and Curran, 2020).

Reflecting previous research where between-domain differences have been observed in the levels of and relationships between motivational phenomena (e.g., Bong, 2001; Bong et al., 2012), the findings of Study 2 showed some differences between the examined subjects. Interestingly, the English course was the only occasion in which connections between intraindividual reward sensitivity and the other variables appeared academically less adaptive (i.e., direct negative prediction on course grade), suggesting that enjoyment of novelty and experiencing one’s successes as rewarding may not always be fully supportive of successfully grasping course content. English was an exception also in that it was the only subject in which previous achievement was uncorrelated with course effort. It is possible that English, being an international lingua franca also in popular culture, is something students are interested in, value, want to learn and achieve highly at, but in which they do not necessarily see academic-style studying, progressing in a structured way and requiring effort, as important, given they are surrounded by the language in popular culture. Frustration or disappointment may ensue, when such structured studying is demanded – or indeed, if achievements do not then reach expectations. Furthermore, while interest is usually associated with positive affect (Hidi and Renninger, 2006), course interest was a negative predictor of course satisfaction in Swedish. It may be that in this subject, students with higher interest in the course content also set higher initial expectations for their success than subsequently reached, thus resulting in dissatisfaction (see, Boekaerts and Niemivirta, 2000). Why this would be the case particularly in this subject and not the others, however, is difficult to discern from the present data. One might speculate that the special position of Swedish as Finland’s second official language, combined with the subject being compulsory, impacts students’ attitudes and interpretations of it in ways that do not apply to the other subjects. Also, while in both studies, the model explained more of the variance in interest and strain, and less in effort, in Study 2, somewhat less variance was explained in Finnish than in the other subjects; further, in Finnish, there were also overall noticeably fewer significant correlations between temperament and the other independent and dependent variables. It appears possible that the motivational appraisals related to studying the mother tongue are more to do with factors not included in this study.

Other subject-specific effects that remain difficult to interpret without further information about, for instance, course content or practices include the positive, reciprocal relation between strain and effort observed in English, and strain early on in the course being positively related to course satisfaction in Finnish. It may be that for some students, the experienced strain represented a positive challenge, rather than a negative hindrance (see, LePine et al., 2004), or that the classroom atmosphere was perceived more supportive of a positive challenge interpretation (see, Kozusznik et al., 2015). This remains something for future research to examine in more detail.

The factor structure of the temperamental sensitivities was found to differ between the two studies, in that a four-factor structure comprising punishment sensitivity, interindividual reward sensitivity, and two sub-dimensions of intraindividual reward sensitivity was found in Study 1, whereas in Study 2, a three-factor structure with a one-dimensional intraindividual reward sensitivity described the data better. Both structures correspond to our previous work (Rawlings et al., 2017), in that the three-factor model of Study 2 reflects the original conceptualisation of inter- and intraindividual reward sensitivities, and the four-factor model of Study 1 repeats the empirically observed finding of intraindividual reward sensitivity further dividing into sub-dimensions of novelty-seeking and positive expressiveness. At this point, it is difficult to take a firm stance on why the factor structures should differ, and we consider this as an issue to be examined more in the future. However, we note that when ESEM was utilised previously (Rawlings et al., 2017), four factors were discovered; PLS-SEM has not been previously used in this context. It may be that the respective analytical methods, with their inherently somewhat different aims (reproduction of covariance matrix vs. maximising explained variance), are a factor contributing to the different structures. Future research may reveal whether the structural difference is indeed a function of the chosen method of analysis, or whether some other, underlying reasons (e.g., age, educational context, or other factors that might contribute to how the experiences reflected by the facets differentiate) moderate the empirical dimensionality of intraindividual reward sensitivity. Nevertheless, based on the present and previous findings, novelty seeking would seem to be the driving aspect in terms of predictions on motivational appraisals.

Naturally, the present research has some limitations. The mechanisms behind the connections between temperament and motivation are likely to be complex, and situational variables, such as classroom or teacher characteristics (e.g., classroom goal orientation, teacher enthusiasm; Carmichael et al., 2017) are likely to play a part. Future research should take into account, and aim to control the effects of contextual variables that remained unaccounted for in the present study. Furthermore, between-subjects similarities and differences might also be explained by predictors not included here, such as conscientiousness (Trautwein et al., 2009) or students’ differing perceptions of the relative utility value of a given domain or course (Eccles, 2009). It is vital that in future research, such potential other predictors are carefully considered and included in the design, so their impact might be explicitly examined.

Study 1 was conducted cross-sectionally, and in Study 2, the number of participants was quite small, which reduces statistical power and generalisability. The latter is also affected by the sample in Study 2 being socio-culturally relatively homogeneous, although focussing on one age-cohort in one school also enabled reducing the effects of contextual factors, such as differences in school cultures and practices. Also, whilst the relationships in Study 2 were examined longitudinally, the time span was fairly short, and overall, there were fewer predictions than expected. However, as the observed relationships in both studies were mostly in keeping with our assumptions and reflect previous findings, the line of research appears potentially fruitful. Studying these relationships over a longer period of time (e.g., entire school year) as well as on a micro level (e.g., using the Experience-Sampling Method; Csikszentmihalyi and Larson, 2014), in other cultural settings, and whilst taking into account the potential influence brought by differences in participants’ socio-economic backgrounds, might increase understanding of their dynamics and development.

However, overall, the findings of both studies were largely in line with our assumptions, reflect the understanding gained from our previous research, namely, that punishment sensitivity as well as sensitivity to qualitatively different kinds of reward are differentially related to motivation in a learning context (Rawlings et al., 2017), and ultimately suggest that individual sensitivities should be taken into account in classroom practices. While it appears that temperament may indeed be an antecedent to strain (see, Strelau, 2001), it is encouraging that the tendency to focus on, avoid, and withdraw from anxiety-arousing situations appears – at least by the present findings – not to stifle experiencing academic interest or effort exertion. It may indeed be that kindling interest in school subjects might help counteract stress experiences (see, e.g., Silvia, 2017) to which students high in punishment sensitivity or interindividual reward sensitivity seem to be prone. Also, a focus on supporting skill development appears important in this respect, given the inverse links between previous achievement and strain.

## Conclusion

The findings of the two studies suggest that temperamental sensitivity to punishment and qualitatively different kinds of reward may differentially contribute to students’ motivational appraisals, even when accounting for previous skill level and subject interest. The similarities between the two studies as well as across the school subjects thus suggest that students’ individual characteristics may render them more susceptible to certain motivational experiences and educational outcomes even across subject content. However, as differences were also observed in the given connections, future research would likely benefit from taking into consideration other motivational (e.g., utility value, efficacy beliefs, goals) and contextual (e.g., instructional practices, teacher relationships, classroom motivational climate) factors that might have an impact on students’ domain-specific beliefs and course-related appraisals.

That said, by the present results, punishment sensitivity and heightened responsiveness to reward dependent on others’ (perceived) attitudes and actions toward oneself may be related to experiences of psychological strain, whereas positive responsiveness to novelty and own successes may support interest and effort. Although the role of interest in performance remains unsettled, subject interest seems to facilitate course-specific interest appraisals. Further, both interest and sufficient competence may buffer students against feelings of strain, leaving more mental resources available for beneficial learning processes. Given the detrimental effects of strain, attention should be given to its antecedents. In light of the present results, supporting interest development and a focus on the personal meaningfulness of one’s studies rather than on achievement relative to others may be helpful.

## Data Availability Statement

The raw data supporting the conclusions of this article will be made available by the authors, without undue reservation.

## Ethics Statement

Ethical review and approval was not required for the study on human participants in accordance with the local legislation and institutional requirements. Written informed consent to participate in this study was provided by the participants’ legal guardian/next of kin.

## Author Contributions

AR analysed the data and drafted the manuscript. AT contributed to the study design, data collection, and writing of the manuscript. MN outlined the research design, provided support for the data analysis, and contributed to the writing of the manuscript.

## Conflict of Interest

The authors declare that the research was conducted in the absence of any commercial or financial relationships that could be construed as a potential conflict of interest.

## Footnotes

1 Note that for comparison, we ran analyses also with CFA instead of ESEM on the measurement model for temperament. Although the patterning of effects were virtually identical, an inspection of the details of the models supported the use of ESEM. The more restrictive CFA was inferior in handling item-level dependencies within the data, thus resulting in less accurate measurement models (e.g., inflated latent correlations).

2 We ran a series of analyses removing each respective motivational appraisal, in turn and singly, to test for potential sources of confounding. These auxiliary analyses resulted in virtually no changes in the effects of the other appraisals.

3 Typically, over 90% of Finnish students continue in formal upper-secondary education directly after graduating from comprehensive school (on average the year the students turn 16), with approximately half of these students then entering academically focused general upper-secondary schools requiring a nationally set level of GPA, and the other half entering vocational upper-secondary schools providing professional qualifications (Official Statistics of Finland, 2020). The present study is conducted in the more academic general upper-secondary context, and hence, our participants are a sample of this perhaps somewhat more academically oriented half of Finnish adolescents.
