# Supplementary material for: Temperamental Sensitivities Differentially Linked With Interest, Strain, and Effort Appraisals
Source: Front Psychol. 2021 Jan 8;11:551806. doi: 10.3389/fpsyg.2020.551806 (PMC7820763; doi:10.3389/fpsyg.2020.551806)
Supplement: Supplementary file 1 [file Data_Sheet_1.pdf]

SUPPLEMENTARY APPENDIX A.

Factor Loadings and Explained Variance (ESEM) of Temperamental Sensitivity Items (Study 1).

Factors and Items	*1*	*2*	*3*	*4*	R^2
1 PUNISHMENT SENSITIVITY					
I am easily shy in the company of people I don't know and in new situations.	**.741**	-.058	*.147*	-.029	.498
I feel very uncomfortable in new situations and places.	**.726**	.118	-.046	-.069	.586
I prefer to withdraw in situations that feel unpleasant or difficult.	**.649**	.027	.038	*.189*	.397
I get upset easily if I am criticised or told off.	**.505**	.121	-.036	*.218*	.294
I don't like talking or performing in public (e.g., answering in class).	**.504**	*-.171*	-.076	.014	.321
2 INTERINDIVIDUAL REWARD SENSITIVITY					
I often do things just to be praised (e.g., by the teacher).	-.066	**.689**	-.028	-.119	.434
I often aim to impress other people.	.103	**.625**	-.047	.033	.405
I will gladly be the centre of attention.	.080	**.524**	*.236*	.036	.389
I sometimes act hastily just to get a reward or praise.	*-.175*	**.386**	.102	*.212*	.348
3 INTRAINDIVIDUAL REWARD SENSITIVITY (NOVELTY-SEEKING)					
I find new things exciting.	.016	-.103	**.848**	.018	.693
I think it is exciting to get into new and surprising situations.	-.083	.033	**.684**	-.087	.493
I will readily seek out novel situations.	**-.341**	.081	**.401**	.127	.474
4 INTRAINDIVIDUAL REWARD SENSITIVITY (POSITIVE EXPRESSIVENESS)					
I don't hold back my joy and enthusiasm when something nice happens to me.	-.025	-.068	-.047	**.826**	.639
I express my excitement and enjoyment openly, when I succeed at something.	-.001	.133	.103	**.615**	.502

Note. Factor loadings significant at $p < .001$ given in bold, and $p < .05$ in italics. All R^2 significant at $p < .001$.

Factor Loadings and Explained Variance (CFA) of Motivational Appraisal Items (Study 1).

Factors and Items	*1*	*2*	*3*	R^2
1 MATHEMATICS INTEREST				
I am interested in math. [a]	.910			.828
Math is a personally important subject for me. [b]	.848			.719
I look forward to math classes a lot. [b]	.811			.658
I would like to learn more about some of the things we do in math classes. [a]	.757			.572
It is important for me to understand math. [c]	.721			.520
I like doing math things also in my spare time. [a]	.718			.515
I enjoy challenging math tasks. [c]	.567	-.395		.705
I sometimes look at math-related web sites in my spare time. [a]	.495			.245
2 MATHEMATICS STRAIN				
The topics and tasks in math are really demanding		.732		.536
Math classes are really exhausting		.701		.491
Studying math stresses me out.		.634		.401
3 MATHEMATICS EFFORT				
I always try my best in math classes. [d]			.802	.644
I put serious effort into solving the math problems we do in class. [e]			.802	.643
I always try to solve all problems we have for homework in math. [d]			.791	.626

Note. All factor loadings and R^2 significant at $p < .001$. Item sources: [a]Frenzel, Goetz, Pekrun, & Watt (2010); [b]Marsh, Trautwein, Lüdtke, Köller, & Baumert (2005); [c]Gottfried (1985); [d]Trautwein et al. (2015); [e]Trautwein, Lüdtke, Roberts, Schnyder, & Niggli (2009).

SUPPLEMENTARY APPENDIX B.

Bootstrapped Factor Loadings of Latent Variables (Study 2).

Factors and Items	Finnish (N = 140)	Swedish (N = 124)	English (N = 141)	Mathematics (N = 81)
PUNISHMENT SENSITIVITY				
I feel very uncomfortable in new situations and places.	.76	.71	.77	.75
I am easily shy in the company of people I don't know and in new situations.	.68	.71	.67	.74
I avoid talking or performing in public (e.g., at lectures).	.69	.70	.72	.79
I get upset easily if I am criticized or told off.	.55[b]	.66	.59[b]	.68
INTERINDIVIDUAL REWARD SENSITIVITY				
I often do things just to be praised.	.74	.71	.76	.74
I will gladly be the centre of attention.	.54	.65	.60	.73
I sometimes act hastily just to get an immediate reward or praise.	.72	.85	.82	.72
INTRAINDIVIDUAL REWARD SENSITIVITY				
I don't hold back my joy and enthusiasm when something nice happens to me.	.71	.74	.72	.75
I express my excitement and enjoyment openly, when I succeed at something.	.75	.75	.72	.75
I will readily seek out novel situations.	.71	.78	.73	.64
I get excited about new things easily.	.80	.76	.81	.66
COURSE INTEREST[a]				
This course is/has been interesting.	.92/.89	.90/.91	.91/.92	.91/.92
The contents of the course are/have been interesting.	.91/.89	.91/.93	.92/.91	.91/.89
The topics of the course are/have been really boring. (r)	.77/.79	.74/.76	.61/.83	.80/.83
COURSE STRAIN[a]				
The topics and tasks of the course are/have been really difficult.	.84/.80	.85/.81	.85/.89	.89/.92
Studying on this course is/has been really exhausting.	.81/.78	.73/.84	.72/.85	.76/.83
This course stresses/has stressed me.	.61/.74	.75/.79	.82/.87	.84/.79
The course is/has been easy. (r)	.57/.74	.80/.88	.86/.86	.87/.86
COURSE EFFORT[a]				
I work/have worked hard to learn what is covered in the course.	.81/.82	.70/.79	.82/.90	.55[b]/.62[b]
I always do/have always done all course homework.	.76/.76	.83/.84	.57/.62	.67[b]/.78
I put/have put serious effort into completing the course.	.87/.88	.86/.83	.82/.90	.78/.85

Note. [a]Time 1/time 2. [b]Item loading not significant at 95% CI, retained within latent variable as DG ρ satisfactory and AVE > .5. (r) reverse-coded

Latent Correlations and Square Root of Average Variance Extracted of Variables (Finnish, N =140; Study 2).

Variable	*1*	*2*	*3*	*4*	*5*	*6*	*7*	*8*	*9*	*10*	*11*	*12*
1 SP	.734											
2 SRinter	.11	.718										
3 SRintra	-.43	.29	.756									
4 Subject Interest	-.05	-.00	.13	-								
5 Previous Achievement	.01	-.07	-.05	.34	-							
6 Course Interest t1	-.16	-.02	.21	.45	.21	.872						
7 Course Strain t1	.13	.18	-.01	-.23	-.15	-.42	.723					
8 Course Effort t1	-.09	-.04	.19	.24	.11	.53	-.21	.815				
9 Course Interest t2	-.14	-.02	.27	.41	.15	.70	-.36	.38	.855			
10 Course Strain t2	.15	.21	.05	-.18	-.06	-.12	.55	-.05	-.22	.722		
11 Course Effort t2	-.09	.09	.26	.22	.14	.42	-.19	.71	.42	.05	.822	
12 Course Grade	.06	-.11	-.09	.14	.56	.03	-.09	.07	.07	-.11	.06	-
13 Course Satisfaction	-.02	-.10	-.14	.06	.28	-.11	.05	.02	-.08	-.18	-.05	.48

Note. Correlations ≥ |.17| are significant at $p < .05$. SP = Sensitivity to Punishment; SRinter = Interindividual Reward Sensitivity; SRintra = Intraindividual Reward Sensitivity. The square root of AVE is given on the diagonal.

Latent Correlations and Square Root of Average Variance Extracted of Variables (Swedish, N = 124; Study 2).

Variable	*1*	*2*	*3*	*4*	*5*	*6*	*7*	*8*	*9*	*10*	*11*	*12*
1 SP	.711											
2 SRinter	.11	.758										
3 SRintra	-.34	.35	.777									
4 Subject Interest	.13	-.02	.07	-								
5 Previous Achievement	.15	-.19	-.19	.52	-							
6 Course Interest t1	.06	.09	.19	.54	.36	.856						
7 Course Strain t1	.02	.21	-.01	-.50	-.46	-.49	.785					
8 Course Effort t1	-.05	-.11	.12	.18	.23	.61	-.21	.803				
9 Course Interest t2	-.07	.08	.25	.51	.26	.79	-.48	.54	.873			
10 Course Strain t2	-.04	.24	.10	-.42	-.47	-.40	.79	-.15	-.40	.829		
11 Course Effort t2	-.14	-.13	.18	.17	.20	.50	-.18	.79	.49	-.15	.826	
12 Course Grade	.23	-.13	-.24	.50	.80	.33	-.44	.18	.26	-.48	.15	-
13 Course Satisfaction	.04	-.21	-.19	.33	.57	.18	-.39	.18	.22	-.47	.18	.66

Note. Correlations ≥ |.18| are significant at $p < .05$. SP = Sensitivity to Punishment; SRinter = Interindividual Reward Sensitivity; SRintra = Intraindividual Reward Sensitivity. The square root of AVE is given on the diagonal.

Latent Correlations and Square Root of Average Variance Extracted of Variables (English, N = 141; Study 2).

Variable	*1*	*2*	*3*	*4*	*5*	*6*	*7*	*8*	*9*	*10*	*11*	*12*
1 SP	.732											
2 SRinter	.08	.741										
3 SRintra	-.47	.30	.762									
4 Subject Interest	-.06	-.04	.02	-								
5 Previous Achievement	.12	-.18	-.17	.56	-							
6 Course Interest t1	-.13	.03	.07	.46	.21	.831						
7 Course Strain t1	.07	.36	.12	-.51	-.56	-.27	.815					
8 Course Effort t1	-.10	.13	.15	.01	-.11	.44	.18	.762				
9 Course Interest t2	-.09	.06	.14	.47	.28	.72	-.31	.31	.888			
10 Course Strain t2	.01	.31	.16	-.53	-.55	-.23	.84	.26	-.30	.867		
11 Course Effort t2	-.18	.04	.19	.01	-.10	.36	.20	.77	.27	.29	.825	
12 Course Grade	.13	-.21	-.28	.51	.82	.24	-.55	-.13	.27	-.60	-.11	-
13 Course Satisfaction	.07	-.12	-.23	.34	.49	.18	-.38	-.09	.18	-.44	-.14	.67

Note. Correlations ≥ |.17| are significant at $p < .05$. SP = Sensitivity to Punishment; SRinter = Interindividual Reward Sensitivity; SRintra = Intraindividual Reward Sensitivity. The square root of AVE is given on the diagonal.

Latent Correlations and Square Root of Average Variance Extracted of Variables (Mathematics, N =81; Study 2).

Variable	*1*	*2*	*3*	*4*	*5*	*6*	*7*	*8*	*9*	*10*	*11*	*12*
1 SP	.782											
2 SRinter	.06	.763										
3 SRintra	-.43	.32	.733									
4 Subject Interest	-.06	-.04	.32	-								
5 Previous Achievement	-.01	-.24	-.11	.10	-							
6 Course Interest t1	-.20	-.06	.29	.62	.06	.873						
7 Course Strain t1	.09	.24	-.04	-.39	-.39	-.44	.842					
8 Course Effort t1	-.11	-.14	.33	.19	-.11	.43	.09	.746				
9 Course Interest t2	-.14	-.10	.37	.57	-.07	.73	-.29	.52	.880			
10 Course Strain t2	-.04	.28	.11	-.36	-.53	-.44	.79	.06	-.22	.853		
11 Course Effort t2	-.24	-.10	.26	.29	.11	.40	-.13	.65	.39	-.17	.767	
12 Course Grade	.03	-.12	-.04	.15	.69	.17	-.35	.03	.02	-.55	.34	-
13 Course Satisfaction	.11	-.17	-.14	.14	.46	.11	-.48	-.02	.05	-.50	.12	.57

Note. Correlations ≥ |.22| are significant at $p < .05$. SP = Sensitivity to Punishment; SRinter = Interindividual Reward Sensitivity; SRintra = Intraindividual Reward Sensitivity. The square root of AVE is given on the diagonal.

Indirect Effects on Dependent Variables (Study 2).

Predictor	Course Interest t2		Course Strain t2		Course Effort t2		Course Grade		Course Satisfaction	
	β	95% CI	*β*	95% CI	*β*	95% CI	*β*	95% CI	*β*	95% CI
FINNISH (*N* = 140)										
SP	-.02	-.20, .20	.12	-.29, .39	-.01	-.21, .20	.06	-.14, .23	-.06	-.25, .15
SRinter	-.07	-.26, .12	.17	-.03, .35	.05	-.20, .25	-.07	-.22, .08	-.03	-.23, .21
SRintra	.24	.08, .40	.07	-.16, .28	.22	.02, .42	-.00	-.15, .16	-.13	-.32, .07
Subject Interest	.37	.22, .51	-.19	-.34, -.01	.16	-.02, .33	-.05	-.18, .10	-.03	-.18, .13
Pr. Achievement	.03	-.11, .17	.01	-.16, .21	.09	-.09, .26	.57	.45, .69	.28	.11, .44
Course Interest t1							-.14	-.32, .05	-.21	-.41, .01
Course Strain t1							-.06	-.21, .11	.06	-.12, .26
Course Effort t1							.08	-.08, .24	.13	-.07, .30
SWEDISH (*N* = 124)										
SP	-.04	-.21, .15	-.01	-.20, .21	-.04	-.26, .23	.09	-.05, .22	-.06	-.25, .13
SRinter	.02	-.17, .20	.18	.02, .35	-.18	-.38, .07	.03	-.09, .15	-.08	-.27, .10
SRintra	.21	.03, .37	.00	-.18, .15	.27	.08, .46	-.09	-.22, .05	-.11	-.29, .07
Subject Interest	.45	.29, .59	-.25	-.43, -.07	.05	-.18, .26	.12	-.02, .25	.07	-.11, .24
Pr. Achievement	.08	-.09, .26	-.30	-.50, -.12	.20	-.03, .43	.72	.60, .83	.50	.34, .67
Course Interest t1							-.01	-.19, .19	-.19	-.42, .05
Course Strain t1							-.09	-.23, .06	-.19	-.39, .01
Course Effort t1							.01	-.14, .16	.15	-.06, .36
ENGLISH (*N* = 141)										
SP	.01	-.17, .22	.01	-.18, .22	-.12	-.37, .16	-.03	-.17, .11	-.06	-.25, .13
SRinter	.03	-.15, .21	.21	.04, .36	-.02	-.23, .19	-.01	-.13, .09	.02	-.14, .20
SRintra	.15	-.03, .34	.06	-.09, .21	.12	-.11, .32	-.16	-.27, -.06	-.20	-.37, -.03
Subject Interest	.42	.23, .60	-.34	-.50, -.17	.06	-.17, .28	.10	-.01, .21	.12	-.02, .29
Pr. Achievement	.09	-.07, .27	-.31	-.49, -.16	-.10	-.32, .12	.73	.65, .81	.40	.23, .57
Course Interest t1							.10	-.01, .21	.10	-.09, .28
Course Strain t1							-.08	-.19, .03	-.09	-.29, .13
Course Effort t1							-.06	-.17, .06	-.08	-.25, .11
MATHEMATICS (*N* = 81)										
SP	-.02	-.21, .19	-.00	-.26, .29	-.15	-.41, .16	.05	-.15, .25	.07	-.24, .35
SRinter	-.17	-.39, .13	.14	-.10, .35	-.11	-.35, .15	.02	-.17, .19	-.04	-.28, .19
SRintra	.23	-.06, .52	.11	-.22, .39	.22	-.09, .54	.03	-.20, .26	-.08	-.32, .20
Subject Interest	.49	.31, .66	-.36	-.53, -.15	.18	-.13, .46	.09	-.07, .27	.12	-.07, .31
Pr. Achievement	-.14	-.31, .05	-.44	-.57, -.27	-.01	-.36, .26	.68	.53, .80	.42	.20, .61
Course Interest t1							.05	-.23, .34	-.15	-.49, .16
Course Strain t1							-.07	-.29, .16	-.42	-.67, -.20
Course Effort t1							.12	-.33, .40	.17	-.15, .49

Note. SP = Punishment Sensitivity; SRinter = Interindividual Reward Sensitivity; SRintra = Intraindividual Reward Sensitivity; Pr. Achievement = Previous Achievement. CI = confidence interval.

Supplementary Material

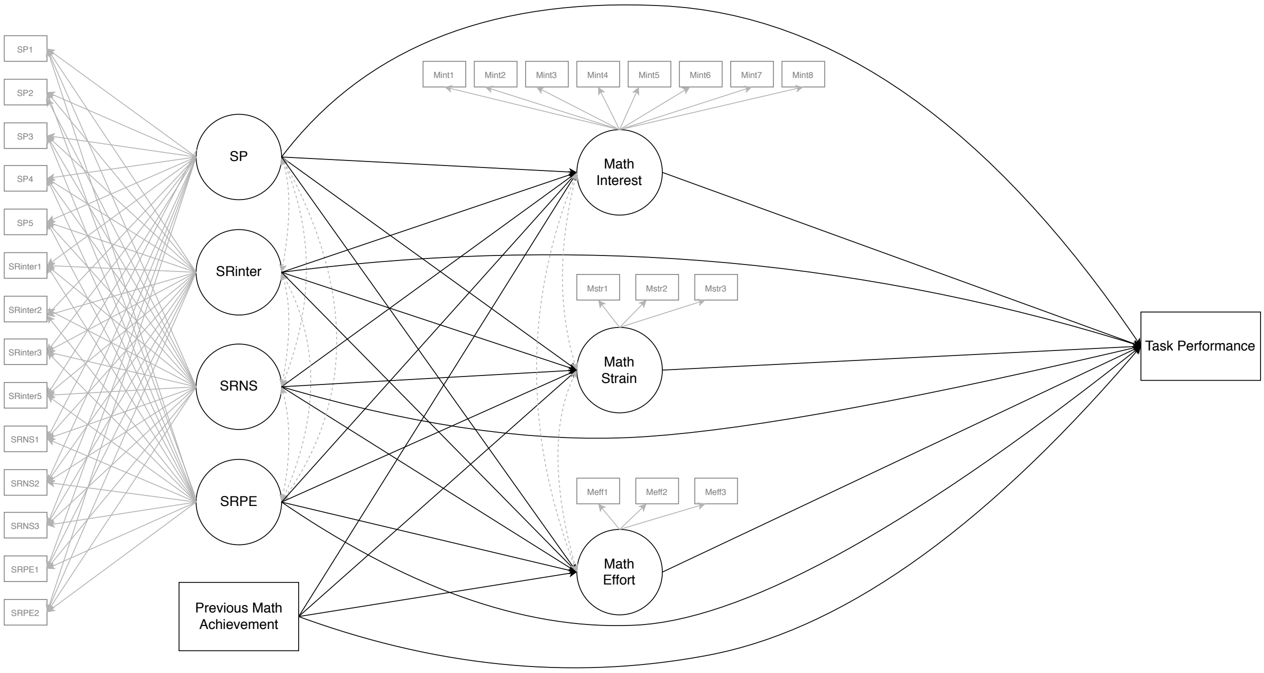

Supplementary Figure 1. Hypothetical model for Study 1. SP = Punishment Sensitivity; SRinter = Interindividual Reward Sensitivity; SRNS = Intraindividual Reward Sensitivity – Novelty-Seeking; SRPE = Intraindividual Reward Sensitivity – Positive Expressiveness.

Measurements and stages of data collection						
**Beginning of school year**		**Early part of course**		**End of course**		**Beginning of next course**
Temperamental sensitivities and subject interests	A	Motivational appraisals (t1)	B	Motivational appraisals (t2)	C	Satisfaction with grades (of the preceding course)

Supplementary Figure 2. Measurements and stages of data collection in Study 2. A = Passage of time varies due to study group differences; B = Passage of time ca. 1.5. months; C = Passage of time ca. 1 week.
